# Assessment of Uncertainties for the NIST 1016 mm Guarded-Hot-Plate Apparatus: Extended Analysis for Low-Density Fibrous-Glass Thermal Insulation

**DOI:** 10.6028/jres.115.004

**Published:** 2010-02-01

**Authors:** Robert R. Zarr

**Affiliations:** National Institute of Standards and Technology, Gaithersburg, MD 20899-8632

**Keywords:** building technology, fibrous glass blanket, guarded hot plate, thermal conductivity, thermal insulation, thermal resistance, uncertainty

## Abstract

An assessment of uncertainties for the National Institute of Standards and Technology (NIST) 1016 mm Guarded-Hot-Plate apparatus is presented. The uncertainties are reported in a format consistent with current NIST policy on the expression of measurement uncertainty. The report describes a procedure for determination of component uncertainties for thermal conductivity and thermal resistance for the apparatus under operation in either the double-sided or single-sided mode of operation. An extensive example for computation of uncertainties for the single-sided mode of operation is provided for a low-density fibrous-glass blanket thermal insulation. For this material, the relative expanded uncertainty for thermal resistance increases from 1 % for a thickness of 25.4 mm to 3 % for a thickness of 228.6 mm. Although these uncertainties have been developed for a particular insulation material, the procedure and, to a lesser extent, the results are applicable to other insulation materials measured at a mean temperature close to 297 K (23.9 °C, 75 °F). The analysis identifies dominant components of uncertainty and, thus, potential areas for future improvement in the measurement process. For the NIST 1016 mm Guarded-Hot-Plate apparatus, considerable improvement, especially at higher values of thermal resistance, may be realized by developing better control strategies for guarding that include better measurement techniques for the guard gap thermopile voltage and the temperature sensors.

## 1. Introduction

In October 1992, NIST officially adopted a new policy [1] for the expression of measurement uncertainty consistent with international practices. The NIST policy is based on recommendations by the Comité International des Poids et Mesures (CIPM) given in the *Guide to the Expression of Uncertainty in Measurement* [2] hereafter, called the GUM.^1^ This report assesses the uncertainties for the NIST 1016 mm Guarded-Hot-Plate apparatus and expresses the uncertainties in a manner consistent with NIST policy. The uncertainty assessment presented herein elaborates on a previous effort [3] presented in 1997 for the production of NIST Standard Reference Material (SRM) 1450c and supersedes the previous error analysis prepared by Rennex in 1983 [4]. Technical details of the apparatus design and fabrication have been described previously [5–6] and, therefore, are only briefly presented here.

The guarded-hot-plate method was standardized in 1945 after many years of effort and designated ASTM Test Method C 177 [7]. Essentially, the method establishes steady-state heat flow through flat homogeneous slabs—the surfaces of which are in contact with adjoining parallel boundaries (i.e., plates) maintained at constant temperatures. The method is considered an absolute measurement procedure because the resulting heat transmission coefficients are directly determined. That is, the test results are not determined by ratio of quantities. In principle, the method can be used over a range of temperatures but, in this report, the mean temperature is limited primarily to 297 K (23.9 °C, 75 °F). This report discusses the measurement principle and presents a procedure for the assessment of uncertainties for a particular lot of low-density fibrous-glass thermal insulation maintained by the NIST Building and Fire Research Laboratory (BFRL).

## 2. Reference Material

The reference material of interest in this report is a low-density fibrous-glass blanket having a nominal bulk density of 9.6 kg · m^−3^ (0.6 lb · ft^−3^). The material lots were manufactured in July 1980 in the form of large sheets (1.2 m by 2.4 m) at nominal thicknesses of 28 mm and 81 mm. After receipt and preparation of the material, the National Bureau of Standards^2^ announced in December 1980 a program [8] to provide thick “calibration transfer specimens” (CTS) on request for use in conjunction with the “representative thickness” provision of the U.S. Federal Trade Commission (FTC) rules published in 1979 [9] hereafter, called the “R-value Rule.” The specimens were 610 mm square and were originally issued at thicknesses of 25 mm, 75 mm, or 150 mm (two 75 mm specimens stacked). Recently, however, in order to satisfy more stringent energy efficiency requirements mandated in U.S. building codes, insulation manufacturers have begun requesting CTS at thicknesses up to 225 mm (three 75 mm specimens stacked). In accordance with test guidelines in the R-value Rule, measurements for customers are usually conducted at a mean temperature of 297 K and a temperature difference of either 22.2 K or 27.8 K (40 °F or 50 °F, respectively) across the specimen [9].

## 3. Steady-State Thermal Transmission Properties

ASTM Practice C 1045 [10] provides a uniform calculation procedure for thermal transmission properties of materials based on measurements from steady-state one dimensional methods such as ASTM Test Method C 177. Table 1 summarizes the generalized one-dimensional equations for thermal resistance (*R*), conductance (*C*), resistivity (*r*), and conductivity (*λ*).

Here, *Q* is the time-rate of one-dimensional heat flow (in units of watts, W) through the meter area of the guarded-hot-plate apparatus, *A* is the meter area of the apparatus normal to the heat flow direction (in units of square meters, m^2^), Δ*T* is the temperature difference across the specimen (in units of kelvins, K), and *L* is the specimen thickness (in units of meters, m). As a rule, NIST provides value assignments and uncertainty for only *R* and, to a lesser extent, *λ* for thermal insulation reference materials. Consequently, this paper presents uncertainty assessments only for thermal resistance (*R*) and thermal conductivity (*λ*).

## 4. Measurement Principle

A guarded-hot-plate apparatus having appropriate plate temperature controllers can be operated in either a double sided mode or in a single-sided mode (also known as two-sided or one-sided mode, respectively). In principle, both modes of operation are covered in Test Method C 177; however, additional information on the single-sided mode is available in ASTM Practice C 1044 [11]. For completeness, this report presents both modes of operation but only the single-sided mode is examined in the uncertainty analysis.

### Double-Sided Mode

**Fig. 1:**
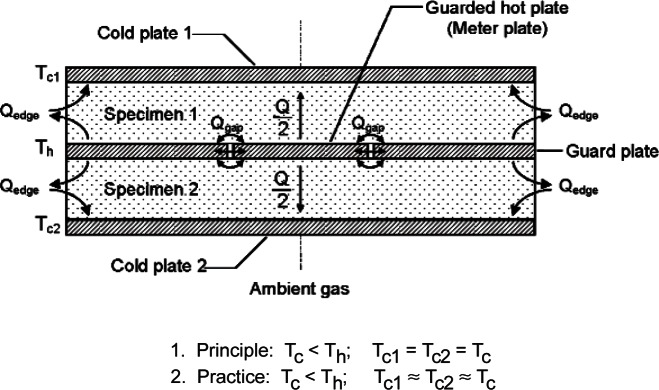
Guarded-hot-plate schematic, double-sided mode of operation—vertical heat flow.

Figure 1 shows the essential features of a guarded-hot-plate apparatus designed for operation near ambient temperature conditions. The plates are shown in a horizontal configuration with heat flow (*Q*) in the vertical (up/down) direction through the specimens. The apparatus is cylindrically symmetric about the axis indicated in Fig. 1. In the traditional double-sided mode of operation, specimens of the same material having nearly the same density, size, and thickness are placed on each surface of the guarded hot plate and clamped securely by the cold plates. Ideally, the guarded hot plate and the cold plates provide constant-temperature boundary conditions to the specimen surfaces. Ideally, lateral heat flows (*Q*_gap_ and *Q*_edge_) are reduced to negligible proportions with proper guarding and, under steady-state conditions, the apparatus provides one-dimensional heat flow (*Q*) normal to the meter area of the specimen pair. Typically, a secondary guard is provided by an enclosed chamber that conditions the ambient gas (usually air) surrounding the plates to a temperature near to the mean specimen temperature (i.e., average surface temperatures of the hot and cold plates in contact with the specimens).

Under steady-state conditions, the operational definition [10] for the mean (apparent) thermal conductivity^3^ of the specimen pair (*λ*_exp_) is

(1) $$\lambda_{\exp}=\frac{Q}{A[{\left(\Delta T/L\right)}_{1}+{\left(\Delta T/L\right)}_{2}]}$$

where:

*Q* = the time rate of one-dimensional heat flow through the meter area of both specimens and, under ideal conditions, is equal to *Q*_m_, the electrical power input to the meter plate (W);
*A* = the meter area normal to the specimen heat flow (m^2^) (see Appendix A for derivation); and,
(Δ*T*/*L*)_1_ = the ratio of the surface-to-surface temperature difference (*T_h_* − *T_c_*) to the thickness (*L*) for Specimen 1. A similar expression is used for Specimen 2.

For experimental situations where the temperature differences and the specimen thicknesses are nearly the same, respectively, Eq. (1) reduces to

(2) $$\lambda_{\exp}=\frac{QL_{\mathrm{average}}}{2A\Delta T_{\mathrm{average}}}.$$

Using the relationship from Table 1, Eq. (2) can be rewritten to determine the thermal resistance of the specimen pair

(3) $$R=\frac{2A\Delta T_{\mathrm{average}}}{Q}.$$

In the double-sided mode of operation, the thermal transmission properties correspond to an average temperature $\bar{T}$ given by $\bar{T}=(T_{h}+T_{c})/2$.

### Single-Sided Mode

**Fig. 2:**
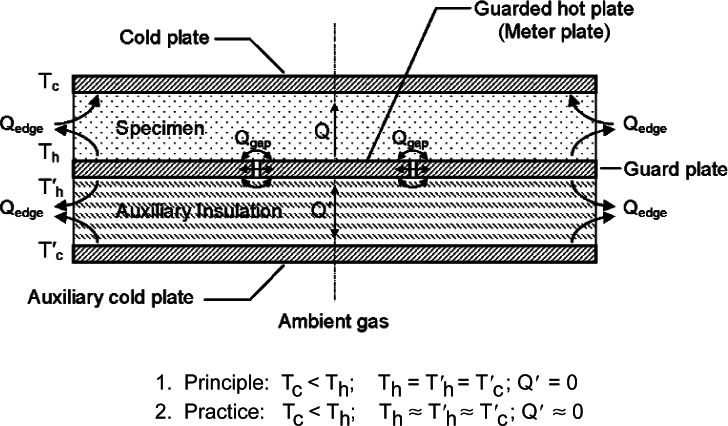
Guarded-hot-plate schematic, single-sided mode of operation—heat flow up.

Figure 2 shows the essential features of a guarded-hot-plate apparatus designed for operation near ambient temperature conditions in the single-sided mode of operation. In the single-sided mode of operation, auxiliary thermal insulation is placed between the hot plate and the auxiliary cold plate, replacing one of the specimens shown in Fig. 1.

The auxiliary cold plate and the hot plate are maintained at essentially the same temperature. The heat flow (*Q*′) through the auxiliary insulation is calculated as follows [11]:

(4) $$Q^{\prime }=C^{\prime }A(T_{h}^{\prime }-T_{c}^{\prime })\approx 0$$

where the prime (′) notation denotes a quantity associated with the auxiliary thermal insulation and *C*′ is the thermal conductance of the auxiliary insulation. The specimen heat flow (*Q*) is computed in the following equation:

(5) $$Q=Q_{m}-Q^{\prime }$$

where *Q_m_* is the power input to the meter plate. Values of *Q*′ are typically less than 1 % of *Q_m_*. For similar materials, *Q* from Eq. (5) is approximately one-half the value obtained for *Q* in Eq. (3) for the double-sided mode.

## 5. Apparatus

**Fig. 3:**
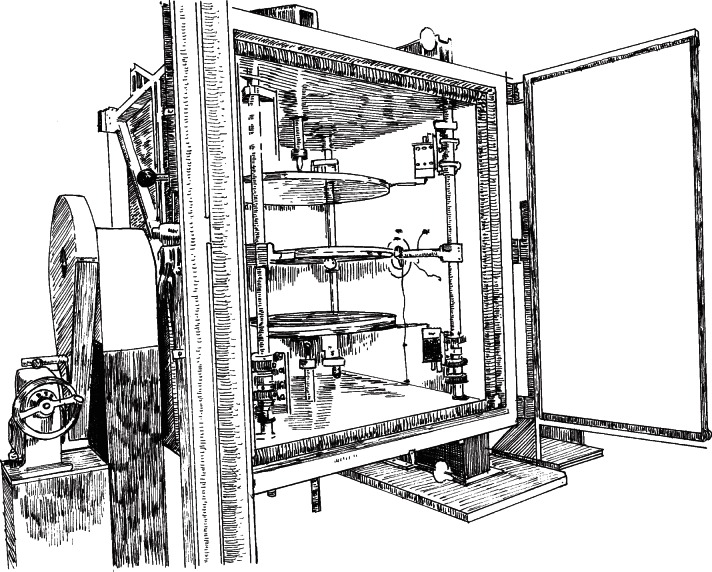
NIST 1016 mm Guarded-Hot-Plate Apparatus.

Figure 3 shows an illustration of the NIST 1016 mm Guarded-Hot-Plate apparatus. The apparatus plates are typically configured in a horizontal orientation and are enclosed by an insulated environmental chamber that can be rotated ± 180°. The plates are made from aluminum alloy 6061-T6. The plate surfaces in contact with the specimens are flat to within 0.05 mm and are anodized black to have a total emittance of 0.89. The hot plate is rigidly mounted on four bearing rods. Each cold plate can translate in the vertical direction for specimen installation and is supported at its geometric center by means of a swivel ball joint that allows the plate to tilt and conform to a nonparallel rigid sample. The clamping force is transmitted axially by extension rods that are driven by a stepper motor and a worm-drive gear. A load cell measures the axial force that the plate exerts on the specimen. The cold plates are constrained in the radial direction by steel cables attached to four spring-loaded bearings that slide on the bearing rods.

### Guarded Hot Plate

The 1016 mm guarded hot plate is nominally 16.1 mm thick and consists of a meter plate^4^ 405.6 mm in diameter and a co-planar, concentric guard plate with an inner diameter of 407.2 mm. The circular gap (also known as “guard gap”) that separates the meter plate and guard plate is 0.89 mm wide at the plate surface. The cross-sectional profile of the gap is diamond shaped in order to minimize lateral heat flow across the gap. The meter plate is supported within the guard plate by three stainless steel pins, equally spaced around the circumference of the meter plate, that are used to adjust the gap to a uniform width and maintain the meter plate in plane with the guard plate. Across its diameter, the meter plate is flat to within 0.025 mm.

The hot-plate heater design, described previously in detail by Hahn et al. [12], utilizes circular line-heat sources located at prescribed radii. The circular line-heat-source for the meter plate is located at a radius of $\surd \bar{2}/2$ times the meter-plate radius which yields a diameter of 287 mm. This location for the heater results in a temperature profile such that the temperature at the gap is equal to the average meter-plate temperature [12]. The heating element is a thin nichrome ribbon filament network, 0.1 mm thick and 4 mm wide, electrically insulated with polyimide, having an electrical resistance at room temperature of approximately 56 Ω.

There are two circular line-heat-sources in the guard plate located at diameters of 524.7 mm and 802.2 mm. The heating elements are in metal-sheathed units, 1.59 mm in diameter, and were pressed in circular grooves cut in the surfaces of the guard plate. The grooves were subsequently filled with a high-temperature epoxy. The electrical resistances at room temperature for the inner and outer guard heaters are approximately 72 Ω and 108 Ω, respectively.

### Meter-Plate Electrical Power

**Fig. 4:**
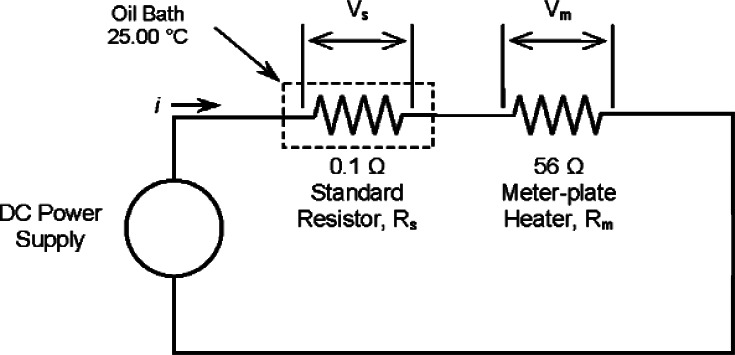
Electrical schematic for meter-plate power measurement.

Figure 4 shows the electrical circuit schematic for the meter-plate power measurement which consists of a four-terminal standard resistor, nominally 0.1 Ω, in series with the meter-plate heater. A direct-current power supply (40 V) provides current (*i*) to the circuit which is determined by measuring the voltage drop (*V_s_*) across the standard resistor (Fig. 4) placed in an oil bath at 25.00 °C. The voltage across the meter-plate heater (*V_m_*) is measured with voltage taps welded to the heater leads in the center of the gap (described above). The meter plate power (*Q_m_*) is the product of *V_m_* and *i*.

### Cold Plates

The cold plates are fabricated from 6061-T6 aluminum and contain channels that circulate an ethylene glycol/distilled water solution. Each plate is 25.4 mm thick and consists of a 6.35 mm thick cover plate bonded with epoxy to a 19.05 mm thick base plate. The base plate has milled grooves 9.5 mm deep and 19.1 mm wide arranged in a double-spiral configuration. This arrangement forms a counter-flow heat exchanger, that is, the supply coolant flows next to the return coolant providing a uniform temperature distribution over the cold-plate surface. The temperature of each cold plate is maintained by circulating liquid coolant from a dedicated refrigerated bath regulated to within ± 0.05 K over a temperature range of −20 °C to 60 °C. The outer surfaces and edges of the cold plates are insulated with 102 mm of extruded polystyrene foam.

### Environmental Chamber

The environmental chamber is a large rectangular compartment having inside dimensions of 1.40 m square by 1.60 m high supported by a horizontal axle on rotational rollers that allow the apparatus to pivot by ± 180° (Fig. 3). Access to the plates and specimens is permitted by front-and-back double-doors. Air is circulated by a small fan in the chamber and is conditioned by a small cooling coil/reheat system located within the chamber. The air temperature ranges from about 5 °C to 60 °C and is maintained to within ± 0.5 K by using the average of five Type T thermocouples located in the chamber.

### Primary Temperature Sensors

The primary temperature sensors are small capsule platinum resistance thermometers (PRTs). The sensor construction is a strain-free platinum element supported in a gold-plated copper cylinder 3.18 mm in diameter by 9.7 mm long backfilled with helium gas and hermetically sealed. The sensors are designed for temperatures from 13 K to 533 K (−260 °C to 260 °C) and the nominal resistance is 100 Ω at 0 °C. The electrical resistance of each 4-wire PRT is measured with a digital multimeter (DMM) that is part of an automated data acquisition system.

Figure 5 shows the locations of the PRTs in the cold plates and hot plate. The cold plate PRT is inserted in a 3.26 mm diameter hole, 457 mm long, bored into the side of the cold plate (Fig. 5a). The hot plate PRT is located in the guard gap at an angle of 69° from the location where meter plate heater wires cross the guard gap (Fig. 5b) based on the theoretical temperature distribution *T*(*r*, θ) determined by Hahn et al. [12] for a similar apparatus. The sensor is fastened with a small bracket on the meter side of the gap at the mid-plane of the plate (*z* = 0) as illustrated in Fig. 5c. The radius to the center of the PRT was computed to be 199.3 mm.

### Temperature Sensors in the Guard Gap

The temperature difference across the guard gap (Δ*T_gap_*) is estimated using an eight junction (4 pairs) Type E^5^ thermopile. The thermopile was constructed from No. 30 AWG (American wire gauge) insulated thermocouple wire 0.25 mm in diameter welded in an argon atmosphere to form small bead junctions. The wire lengths were taken from spools of wire that were scanned using a large temperature gradient (i.e., a bath of liquid nitrogen) to isolate inhomogeneities in the wire. The wire passed from ambient to liquid nitrogen temperature and back to ambient; sections that gave thermoelectric voltages larger than 3 μV for EP wire and 1.7 μV for EN wire were discarded.

**Fig. 6:**
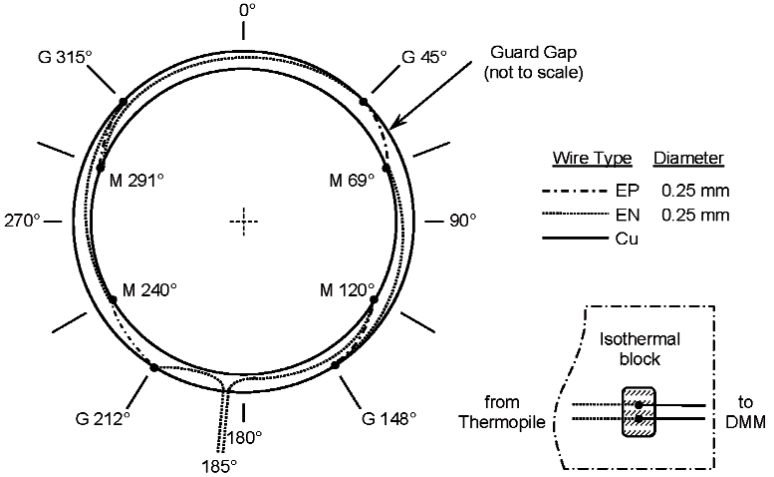
Angular locations of Type E thermopile junctions in the guard gap (not to scale).

Figure 6 shows the angular locations for the individual junctions in the guard gap. The reference angle of 0° is the location where the meter-plate heater leads cross the gap (the same as Fig. 5b).

The thermocouple beads are installed in brackets with a thermally conductive epoxy and fastened, in alternating sequence, to either the meter plate or the guard plate similarly to the method used for the meter-plate PRT (Fig. 5c). Like the PRT, the junctions are located at the mid-plane of the hot plate (that is, *z* = 0 in the axial direction). The EN leads of the thermopile depart the guard gap at an angle of 185° (as shown in Fig. 6) and are connected to copper leads on an isothermal block mounted inside a small aluminum enclosure. The aluminum enclosure is located inside the environmental chamber surrounding the apparatus plates.

### Temperature Control

The three heaters in the guarded hot plate are controlled by a digital proportional, integral (PI) control algorithm that operates by actively controlling the plate temperatures. In other words, the power level is not fixed at a specific level which could lead to temperature drift. Under steady-state conditions, the meter plate temperature is controlled to within ± 0.003 K.

## 6. Measurement Uncertainty Estimation

This section summarizes relevant uncertainty terminology consistent with current international guidelines [1–2] and presents a procedure for the estimation of measurement uncertainty based on practical experiences by analytical chemical laboratories [13]. Using this procedure, an example is given for computation of the measurement uncertainty of the low-density fibrous-glass thermal insulation issued by NIST as a CTS.

### Terminology

The ***combined standard uncertainty*** of a measurement result, *u_c_*(*y*) is expressed as the positive square root of the combined variance $u_{c}^{2}(y)$^2^:

(6) $$u_{c}(y)=\sqrt{\sum_{i-1}^{N}c_{i}u^{2}(x_{i}).}$$

Equation (6) is commonly referred to as the “*law of propagation of uncertainty*” or the “root-sum-of-squares.” The sensitivity coefficients (*c_i_*) are equal to the partial derivative of an input quantity (∂*f* / ∂*X_i_*) evaluated for the input quantity equal to an input estimate (*X_i_* = *x_i_*). The corresponding term, *u*(*x_i_*), is the standard uncertainty associated with the input estimate *x_i_*. The ***relative combined standard uncertainty*** is defined as follows (where *y* ≠ 0):

$$u_{c,r}(y)=\frac{u_{c}(y)}{\left|y\right|}.$$

Each *u*(*x_i_*) is evaluated as either a ***Type A*** or a ***Type B*** standard uncertainty. Type A standard uncertainties are evaluated by statistical means. The evaluation of uncertainty by means other than a statistical analysis of a series of observations is termed a Type B evaluation [1]. Type B evaluations are usually based on scientific judgment and may include measurement data from another experiment, experience, a calibration certificate, manufacturer specification, or other means as described in Refs. [1–2]. It should be emphasized that the designations “A” and “B” apply to the two methods of evaluation, *not* the type of error. In other words, the designations “A” and“B” have nothing to do with the traditional terms “random” or “systematic.” Categorizing the evaluation of uncertainties as Type A or Type B is a matter of convenience, since both are based on probability distributions^6^ and are combined equivalently. Thus, Eq. (6) can be expressed in simplified form as:

(7) $$u_{c}=\sqrt{u_{A}^{2}+u_{B}^{2}}.$$

Examples of Type A and Type B evaluations are provided in Refs. [1–2]. A typical example of a Type A evaluation entails repeated observations. Consider an input quantity *X_i_* determined from *n* independent observations obtained under the same conditions. In this case, the input estimate *x_i_* is the sample mean determined from

$$x_{i}={\bar{X}}_{i}=\frac{1}{n}\sum_{k=1}^{n}X_{i,k}.$$

The standard uncertainty, *u* (*x_i_*) associated with *x_i_* is the estimated standard deviation of the sample mean (where *s* is the standard deviation of *n* observations):

(8) $$u(x_{i})=s({\bar{X}}_{i})=\frac{s}{\sqrt{n}}.$$

The ***expanded uncertainty***, *U*, is obtained by multiplying the combined standard uncertainty, *u_c_* (*y*), by a coverage factor, *k* when an additional level of uncertainty is required that provides an interval (similar to a confidence interval, for example):

(9) $$U=ku_{c}(y)=\sqrt{\sum c_{i}^{2}u^{2}{\left(x_{i}\right)}_{A}+\sum c_{i}^{2}u^{2}{\left(x_{i}\right)}_{B}}.$$

The value of *k* is chosen based on the desired level of confidence to be associated with the interval defined by *U* and typically ranges from 2 to 3. Under a wide variety of circumstances, a coverage factor of *k* = 2 defines an interval having a level of confidence of about 95 % and *k* = 3 defines an interval having a level of confidence greater than 99 %. At NIST, a coverage factor of *k* = 2 is used, by convention [1]. The ***relative expanded uncertainty*** is defined as follows (where *y* ≠ 0):

$$U_{r}=\frac{U}{\left|y\right|}.$$

For Type A evaluations, the ***degrees of freedom***, *ν*, is equal to *n* − 1 for the simple case given in Eq. (8). For the case when *u_c_* is the sum of two or more variance components, an *effective* degrees of freedom is obtained from the Welch-Satterthwaite formula as described in Refs. [1–2]. For Type B evaluations, *ν* is assumed to be infinity. As will be shown later in this report, the Type B evaluation is the dominant component of uncertainty. Therefore, values for *ν* are not necessary and are not ultimately used in determination of the coverage factor, *k*.

### Procedure

The EURACHEM/CITAC Guide [13] provides a practical guide for the estimation of measurement uncertainty based on the approach presented in the GUM [2]. Although developed primarily for analytical chemical measurements, the concepts of the URACHEM/CITAC Guide are applicable to other fields. The primary steps are summarized below:

- Specification of the mathematical process (measurement) model—clear and unambiguous statement of the measurand, i.e., *Y* = *f* (*X*_1_, *X*_2_, … *X*_N_).
- Identification of uncertainty sources—a comprehensive (although perhaps not exhaustive) list of relevant uncertainty sources. A cause-and-effect diagram is a useful means for assembling this list.
- Quantification of the components of the uncertainty sources—a detailed evaluation of the component uncertainties using Type A and/or Type B evaluations described above (for example, Eq. (8)) or in the GUM.
- Calculation of the combined standard uncertainty—propagate the component uncertainties using the “law of propagation uncertainty” given in Eq. (6).
- Calculation of the expanded uncertainty—using a coverage factor of *k* = 2, compute an interval for the expanded uncertainty given in Eq. (9).

## 7. Mathematical Process Model

Mathematical process models are specified for thermal conductivity (*λ*) and thermal resistance (*R*) as determined using the single-sided mode of operation (Fig. 2). For *λ*, the mathematical process model is given by

(10) $$\begin{array}{l} \lambda_{\exp}=\frac{QL}{A(\Delta T)}=\frac{(Q_{m}-\Delta Q)L}{A(T_{h}-T_{c})}\\ \;=\frac{(Q_{m}-Q_{\mathrm{gap}}-Q^{\prime }-Q_{\varepsilon })L}{A(T_{h}-T_{c})} \end{array}$$

where:

*Q_m_* = power input (W) to the meter plate heater;
Δ*Q* = parasitic heat transfer (W) from the meter area (defined more specifically as *Q_gap_*, *Q*′, and *Q_ε_*);
*Q_gap_* = lateral heat flow (W) across the guard gap (i.e., the airspace separation between the meter plate and guard plate shown in Fig. 2);
*Q*′ = heat flow (W) through the meter section of the auxiliary insulation (Fig. 2);
*Q_ε_* = error due to edge heat transfer (W) (i.e., from *Q_edge_* shown in Fig. 2);
*L* = *in-situ* thickness of the specimen during testing (m);
*A* = meter area normal to *Q*(m^2^);
Δ*T* = specimen temperature difference (K);
*T_h_* = temperature of hot plate (K); and,
*T_c_* = temperature of cold plate (K).

For *R*, the mathematical process model is given by

(11) $$\begin{array}{c} R=\frac{A(\Delta T)}{Q}=\frac{A(T_{h}-T_{c})}{Q_{m}-\Delta Q}=\\ =\frac{A(T_{h}-T_{c})}{Q_{m}-Q_{\mathrm{gap}}-Q^{\prime }-Q_{\varepsilon }}. \end{array}$$

One of the major differences between Eqs. (10) and (11) is the absence of the term for specimen thickness (*L*) in Eq. (11). With regards to sign convention for heat flow (*Q*), heat gain to the meter area is assumed to be positive (+) and heat loss is assumed to be negative (−).

## 8. Sources of Uncertainty

**Fig. 7:**
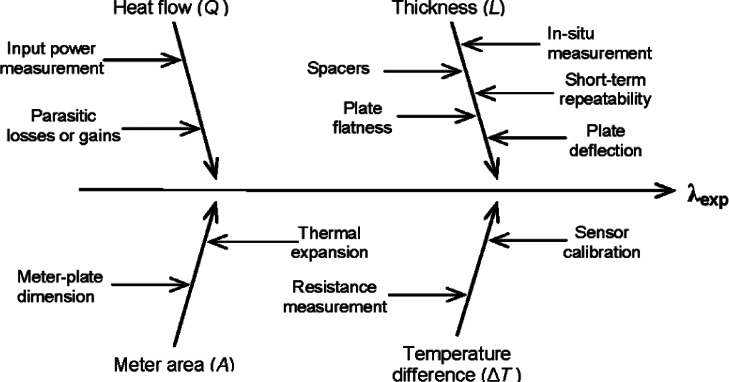
Cause-and-effect chart for *λ*_exp_ (2 levels of contributory effects).

Figure 7 shows a cause-and-effect diagram that has been developed for *λ*_exp_ from Eq. (10). The cause-and-effect diagram is a hierarchical structure that identifies the main sources (shown as arrows directly affecting *λ*_exp_) and secondary factors (shown as arrows affecting *Q*, *L*, *A*, and Δ*T*) of contributory uncertainty. Tertiary (and additional hierarchical) factors of contributory uncertainty are not shown in Fig. 7. In general, the uncertainty sources in Fig. 7 can be grouped in one of three major metrology categories—dimensional metrology for meter area (*A*) and thickness (*L*); thermal metrology for temperature (*T*); and, electrical metrology for voltage (*V*) and resistance (Ω) measurements. The analysis of parasitic heat losses and/or gains (Δ*Q*) requires either additional heat-transfer analyses or experiments (or both).

From Fig. 7, a comprehensive, but not exhaustive, list of uncertainty sources is developed as shown in Table 2. This particular list could be applied to other apparatus but is most applicable to the NIST 1016 mm Guarded-Hot-Plate apparatus for single-sided measurements of low-density fibrous-glass blanket thermal insulation. Other materials, mode of operation, apparatus, etc. may require a (slightly) different listing of sources (see, for example, the uncertainty analysis for NIST SRM 1450c [3]).

The list of contributary sources of uncertainty for *R* is the same as the list given in Table 2 except the contributory source for *L* would be omitted, as shown in Eq. (11).

## 9. Quantification of Uncertainty Components

Analysis of the standard uncertainties for meter area (*A*), thickness (*L*), temperature difference (Δ*T*), and power (*Q*) are presented in this section. A useful approach that is followed in this report is to treat each uncertainty component separately and evaluate the uncertainty component as either a Type A or Type B standard uncertainty [1–2]. The example presented here is for specimens of low-density fibrous-glass thermal insulation taken from the CTS lot of reference material in thicknesses of 25.4 mm, 76.2 mm, 152.4 mm, 228.6 mm. The guarded-hot-plate measurements were conducted at a mean temperature of 297 K and a temperature difference of 22.2 K. The apparatus was operated in the single-sided mode of operation utilizing a specimen of expanded polystyrene foam having a nominal thickness of 100 mm as the auxiliary insulation (Fig. 2).

### Meter Area (A)

The meter area is the mathematical area through which the heat input to the meter plate (*Q*) flows normal to the heat-flow direction under ideal guarding conditions (i.e., *Q_gap_* = *Q_ε_* ≡ 0) into the specimen. It is important to emphasize that the meter area is not the same as the area of the meter plate (shown in Figs. 1 and 2). The circular meter area was calculated from Eq. (12) below (see Appendix A for derivation):

(12) $$A=\frac{\pi }{2}(r_{o}^{2}+r_{i}^{2})(1+\alpha \Delta T_{mp})$$

where:

*r*_o_ = outer radius of meter plate (m);
*r*_i_ = inner radius of guard plate (m);
*α* = coefficient of thermal expansion of aluminum (alloy 6061-T6) (K^−1^); and,
Δ*T_mp_* = temperature difference of the meter plate from ambient (K) = *T_h_* − 20 °C.

The application of Eq. (6) to Eq. (12) yields

$$\begin{array}{l} u_{c}(A)=\\ \sqrt{c_{r_{o}}^{2}u^{2}(r_{o})+c_{r_{i}}^{2}u^{2}(r_{i})+c_{a}^{2}u^{2}(\alpha )+c_{\Delta T_{mp}}^{2}u^{2}(\Delta T_{mp})} \end{array}$$

with

$$\begin{array}{l} c_{r_{o}}=\partial A/\partial r_{o}=\pi r_{o}{\left(1+\alpha \Delta T_{mp}\right)}^{2}\\ c_{r_{i}}=\partial A/\partial r_{i}=\pi r_{i}{\left(1+\alpha \Delta T_{mp}\right)}^{2}\\ c_{\alpha }=\partial A/\partial \alpha =\pi \Delta T_{mp}(r_{o}^{2}+r_{i}^{2})(1+\alpha \Delta T_{mp})\\ c_{\Delta T_{mp}}=\partial A/\partial (\Delta T_{mp})=\pi \alpha (r_{o}^{2}+r_{i}^{2})(1+\alpha \Delta T_{mp}) \end{array}$$

#### Plate Dimensions

The design gap dimensions [5] for the meter plate and the guard plate diameters are 405.64 mm (15.970 in) and 407.42 mm (16.040 in), respectively. In 1994, as part of an extensive sensor calibration check, the meter plate was separated and removed from the guard plate. Using a coordinate measuring machine, the roundness of the meter plate was checked at six locations at the periphery and the diameter was determined to be 405.67 mm (15.971 in). During re-assembly, a uniform gap width of 0.89 mm (0.035 in) was re-established using three pin gages spaced at equiangular intervals between the meter plate and guard plate. The uncertainty of the pin gages was + 0.005 mm / − 0.000 mm. Based on these check measurements, the input values for *r_o_* and *r_i_*, were determined to be 0.20282 m and 0.20371 m, respectively, and the standard uncertainty for both input values was taken to be 0.0254 mm (0.001 in.).

#### Thermal Expansion

For α, an input value of 23.6 × 10^−6^ K^−1^ was taken from handbook data for aluminum alloy 6061-T6. The standard uncertainty for the value of α was assumed to be 10 % (that is, 2.36 × 10^−6^ K^−1^). For tests conducted at a mean temperature of 297 K and a specimen temperature difference of 22.2 K, the meter plate temperature (*T_h_*) was maintained at 308 K(35 °C, 95 °F); thus, Δ*T_mp_* was equal to + 15 K. The standard uncertainty for Δ*T_mp_* was determined to be 0.086 K (and will be discussed later in the section on Δ*T* uncertainty).

#### u_c_(A)

Substituting the above input estimates into Eq. (12), yields a meter area (*A*) of 0.12989 m^2^. For *u_c_*(*A*), the input estimates (*x_i_*), sensitivity coefficients (*c_i_*), standard uncertainties (*u* (*x_i_*)), and evaluation method (Type A or B) are summarized in Table 3. The last column in Table 3 provides values for *c_i_* ·· *u*(*x_i_*) to assess the uncertainty contribution for each input *X_i_*. The combined standard uncertainty *u_c_*(*A*) and relative standard uncertainty *u_c,r_*(*A*) were determined to be 2.4732 × 10^−5^ m^2^ and 0.019 %, respectively. This estimate for *u_c_*(*A*) is quite small near ambient temperature but increases as *T_h_* departs from ambient conditions.

### Thickness (L)

In the single-sided mode of operation, the in-situ thickness of the specimen (Fig. 2) is monitored during a test by averaging four linear position transducers attached to the periphery of the cold plate at approximate 90° intervals.^7^ Each device consists of a digital readout and a slider that translates in close proximity to (but not in contact with) a 580 mm precision tape scale bonded to a precision ground plate of a low thermal expansion iron-nickel (FeNi36) alloy. In operation, the slider is excited with a pair of oscillating voltages which are out-of-phase by 90°. The electrical windings on the scale are inductively coupled with the slider and the resulting output signal from the scale is resolved and processed by the digital readout. As the slider follows the axial movement of the cold plate, the corresponding output signal represents the linear distance between the translating cold plate and the stationary hot plate.

The digital readouts are reset by placing a set of four fused-quartz spacers of known thickness between the cold plate and hot plate. Fused-quartz tubing was selected because of its low coefficient of thermal expansion (5.5 × 10^−7^ K^−1^) and high elastic modulus (72 GPa). The tubes have nominal inner and outer diameters of 22 mm and 25 mm, respectively. Loose-fill thermal insulation was placed in the tubes to suppress any convective heat transfer. Because the fibrous-glass blanket CTS is compressible, the plate separation is maintained during a test by four fused-quartz spacers placed at the periphery of the specimen at the same angular intervals as the four linear position transducers described above. Four sets of spacers having lengths of 25.4 mm, 76.2 mm, 152.4 mm, and 228.6 mm cover the thickness range of interest for fibrous-glass blanket CTS.

The combined standard uncertainty for *L* is given by

(13) $$u_{c}(L)=\sqrt{u^{2}(L_{1})+u^{2}(L_{2})+u^{2}(L_{3})+u^{2}(L_{4})+u^{2}(L_{5})}$$

where the sensitivity coefficients are equal to unity (*c_Li_* = 1) and the contributory uncertainties, identified in Fig. 7, are

*u*(*L*_1_) = standard uncertainty of the in-situ linear position measurement (m);
*u*(*L*_2_) = standard uncertainty of the fused-quartz spacers (m);
*u*(*L*_3_) = standard uncertainty of the repeatability of the linear position measurement (m);
*u*(*L*_4_) = standard uncertainty of the plate flatness (m); and,
*u*(*L*_5_) = standard uncertainty of the cold plate deflection under axial loading (m).

The contributory uncertainties *u*(*L_i_*) are discussed in detail below.

#### u(L_1_)—In-situ Measurement

During a test, the digital readouts are recorded manually and the estimate for *x*(*L*_1_) is determined from the sample mean of the four observations. Two contributory effects comprise *u*(*L*_1_): 1) multiple observations (Type A evaluation); and, 2) the measurement system uncertainty (Type B evaluation). Thermal expansion effects of the linear tape scales were neglected because the iron-nickel (36 %) alloy has a low coefficient of thermal expansion and the tests are conducted near ambient conditions of 297 K. Equation (8) is applied to evaluate the Type A standard uncertainty where *s* is the standard deviation of the four transducers (*n* = 4). The Type B evaluation is the uncertainty specification stated by manufacturer (*k* = 1) of 0.005 mm. Application of Eq. (7) yields

$$u_{c}(L_{1})=\sqrt{u_{A}^{2}+{\left(5.0\times 10^{-6}\right)}^{2}}$$

where *u_A_* varies for a particular test. Estimates for *u* (*L*_1_)*_A_* for a test thickness of 25.4 mm are summarized at the end of this section (see Table 5).

#### u(L_2_)—Spacers

Two contributory effects comprise *u*(*L*_2_): 1) multiple length observations (Type A evaluation); and, 2) caliper uncertainty (Type B evaluation). Thermal expansion effects were neglected because fused quartz has a low coefficient of thermal expansion (5.5 × 10^−7^ K^−1^) and the tests were conducted near ambient conditions of 297 K. Deformation of the spacers under load was also neglected because of the cross-sectional area of tubing and the relatively high value for elastic modulus. The length of each spacer was measured under ambient conditions with digital calipers and *x*(*L*_2_) was determined from the sample mean of four observations. Equation (8) is applied to evaluate the Type A standard uncertainty where s is the standard deviation of the four observations (*n* = 4). The Type B evaluation assumes a uniform distribution with an interval of 2a [2]; thus, $u_{B}=a/\surd \bar{3}$ where *a* is the smallest length interval of the caliper. The estimates for *u_A_* and *u_B_* vary for each set of spacers and for the type of measurement calipers, respectively. Estimates for *u*(*L*_2_)*_A,B_* for a test thickness of 25.4 mm are summarized at the end of this section (see Table 5).

#### u(L_3_)—Repeatability

The short-term repeatability of the linear position transducers was determined from a series of replicate measurements. For these measurements, the digital readouts were initially set to the length values of each set of fused-quartz spacers placed between the cold plate and hot plate. The cold plate was lifted from the spacers and subsequently lowered in contact with the spacers five times to check within-day variation. The procedure was repeated for four consecutive days to check the day-to-day variation (20 observations total).

The standard uncertainty for *u*(*L*_3_) was determined using the Type A evaluation given in Eq. (14) [14]

(14) $$u(L_{3})=\sqrt{s_{a}^{2}+\left(\frac{r-1}{r}\right)s_{d}^{2}};$$

where *s_a_* is the standard deviation of the daily averages (between-day variation), *s_d_* is the (pooled) within-day standard deviation, and *r* is number of replicates per day (*r* = 5). Table 4 summarizes replication statistics for nominal specimen thicknesses of 25.4 mm, 76.2 mm, 152.4 mm, and 228.6 mm. Values for within-day average and within-day standard deviation for the 5 replicates are given in columns 4 and 5, respectively, and values for *s_a_*, *s_d_*, and *u*(*L*_3_) for each nominal level of thickness are summarized in the last three columns of Table 4. Note that values of *u*(*L*_3_) in Table 4 do not appear to be correlated with *L*. The degrees of freedom (*v*) for Eq. (14) were determined from the Welch-Satterthwaite formula [1] and the value is summarized at the end of this section (see Table 5).

#### u(L_4_)—Plate Flatness

Two contributory effects comprise *u*(*L*_4_): 1) multiple thickness observations (Type A evaluation); and, 2) coordinate measuring machine (CMM) uncertainty (Type B evaluation). As discussed above, the meter plate dimensions were checked with a CMM in 1994. The thickness of the plate was measured at 32 different locations using a CMM and the estimate for *x*(*L*_4_) was determined from the sample mean of 32 observations. The standard deviation (*s*) was 0.0131 mm and, thus, the relative flatness over the meter plate is (0.013 mm)/(406.4 mm) = 0.003 %. It is interesting to note that the flatness specification given in C177-04 is 0.025 % [7]. Application of Eq. (8) to evaluate the Type A standard uncertainty yields:

$$u{\left(L_{4}\right)}_{A}=1.31\times 10^{-5}m/\sqrt{32}=2.32\times 10^{-6}m.$$

The Type B evaluation is the uncertainty specification (*k* = 1) for the CMM of 0.0051 mm. Because the cold plate was fabricated with the same machine finish as the meter plate, the cold plate flatness is assumed to be nearly the same as the meter plate. In this case, Eq. (7) becomes:

$$u_{c}(L_{4})=\sqrt{2(u_{A}^{2}+u_{B}^{2})}.$$

Substituting the values for the Type A and Type B evaluations given above yields a standard uncertainty for *L*_4_ of 0.0079 mm. The value of *u*(*L*_4_) (0.0079 mm) is apparatus dependent and, thus, is fixed for all values of specimen thickness.

#### u(L_5_)—Cold Plate Deflection

The potential deflection of the (large) cold plate under a mechanical load is evaluated as a Type B uncertainty using classical stress and strain formulae developed for flat plates. As will be discussed below, this approach is an approximation. Recall that the clamping force on the specimen and auxiliary insulation is transmitted axially by extension rods (Fig. 3). The axial force is applied over a circular area at the center of each plate and is assumed to be uniformly distributed through a ball joint connection between the plate and extension rod. In the single-sided mode of operation, the auxiliary insulation is a rigid specimen of expanded polystyrene foam which supports the hot plate (Fig. 2). For a uniform load over a concentric circular area of radius *r*, the maximum deflection *y_max_* at the center of the cold plate is given by the following formula from Ref. [15]. In this case, simple edge support is assumed because the test specimen is compressible and the plate separation is maintained by edge spacers.

(15) $$\begin{array}{l} u(L_{5})=y_{\max}=\\ -\frac{3W(m^{2}-1)}{16\pi Em^{2}t^{3}}\left[\frac{(12m+4)a^{2}}{m+1}-4r^{2}\ln\frac{a}{r}-\frac{(7m+3)r^{2}}{m+1}\right] \end{array}$$

where:

*W* = total applied load (N);
*m* = reciprocal of Poisson′s ratio (dimensionless);
*E* = modulus of elasticity (N · m^−2^);
*t* = thickness of the plate (m); and,
*a* = radius of the plate (m).

Based on load cell measurements, a conservative estimate for the net applied force (*W*) for the cold plate was assumed to be 356N (80 lbf). The plate is 1.016 m in diameter and 0.0254 m thick and is fabricated from aluminum alloy 6061-T6. The values for *m*, *E*, and *r* were taken to be (0.33)^−1^ = 3.0, 6.9 × 10^7^ kPa (10 × 10^6^ lb_f_ · in^−2^), and 0.305 m, respectively. Substituting into Eq. (15) yields a value of 0.031 mm for *y_max_*, which is the dominant component of the thickness uncertainty and is essentially fixed for each level of specimen thickness (for constant loading).

In general, the uncertainty due to plate deflection depends on the apparatus plate design (i.e., dimensions and material), the rigidity of the test specimen, and the magnitude and application of the load applied. The major limitations for this assessment approach are:

- The cold plate is not simply supported as assumed in Eq. (15). The plate is actually constrained by the fused-quartz spacers at four locations around the periphery of the plate.
- The cold plate is not a solid plate. As discussed above, the cold plate is actually a composite construction to allow the flow of coolant internally within the plate.

#### u_c_(L)

Table 5 summarizes the sources, sensitivity coefficients (*c_i_*), uncertainty components *u*(*L_i_*), and the evaluation method (Type A or B) for a thickness of 25.4 mm (*L*_25.4_). As described above, the component uncertainties are either test dependent (*u*(*L*_1_)), spacer dependent (*u*(*L*_2_)), process dependent (*u*(*L*_3_)), or apparatus dependent (*u*(*L*_4_)) and *u*(*L*_5_)). The final two components are essentially fixed for all thicknesses. Consequently, only the first three rows of Table 5 are applicable for 25.4 mm thick specimens. Application of Eq. (13) yields a combined standard uncertainty for *L*_25.4_ of 0.038 mm (*u_c,r_* (*L*) = 0.15 %). It is interesting to note that C 177-04 requires that the specimen thickness be determined to within 0.5 % [7].

Table 6 summarizes *u*(*L_i_*), *u_c_*(*L*), and *u_c,r_*(*L*) for specimen thicknesses of 25.4 mm, 76.2 mm, 152.4 mm, and 228.6 mm. As discussed above, the dominant component for all levels of thickness is *u*(*L*_5_), the uncertainty due to potential deflection of the cold plate. As a result, the variation of *u_c_*(*L*) is small over the range of thicknesses. One should note that the values given in Table 6 are valid only for the apparatus described herein. Other guarded-hot-plate apparatus would have different sources and values for the thickness uncertainty components. For example, the uncertainty due to plate flatness could be much larger if proper attention is not given to the plate design and fabrication.

### Temperature Difference (ΔT)

As discussed above, the *primary* plate temperatures (Figs. 1–2) are monitored during a test by computing temporal averages of three small capsule platinum resistance thermometers (PRTs) (*n* = 240 observations taken over a steady-state interval of 4 h). The uncertainty sources *u*(*T_i_*) for the primary temperature sensors are discussed in detail below. *Secondary* temperature sensors such as thermocouples and thermistors located in the plates, and their corresponding uncertainties, are not discussed because these sensors are not input quantities in the mathematical process models given in Eqs. (10) and (11).

#### u(T_1_)—Measurement

During a typical CTS test (4 h in duration), the electrical resistances of the PRTs are recorded every minute by an automated data acquisition system (*n* = 240). Two major contributory effects comprise *u*(*T*_1_): 1) regression equation coefficients (Type A evaluation); and, 2) the measurement system uncertainty (Type B evaluation). (The standard uncertainty for repeated observations of Δ*T* (Type A evaluation) was less than 0.0002 K and was neglected in further analyses.)

1. For each PRT, individual observations in ohms (Ω) were converted to temperature using a curve fit to the calibration data (discussed below). The curve-fits were obtained using a statistical plotting package from NIST. The residual standard deviation for the fit of each set of calibration data was “pooled” and the resulting standard uncertainty is 0.0052 K. The degrees of freedom from the regression analyses were aggregated for a value of 15.
2. The Type B standard uncertainty for the resistance measurement assumes a uniform distribution with an interval 2*a* [2] where *a* was determined from the specification of the manufacturer for the digital multimeter (DMM). For a = 0.039 Ω at the 300 Ω DMM range, $u_{B}=a/\surd \bar{3}$ = 0.022 Ω. This standard uncertainty in ohms was propagated using the above curve fit to yield a standard uncertainty for temperature of 0.058 K.

#### u(T_2_)—Calibration

The PRTs were calibrated by the NIST Thermometry Group by comparison with a standard platinum resistance thermometer in stirred liquid baths. The thermometer was inserted into a test tube partially filled with mineral oil which, in turn, was placed in the calibration bath. In 1981, the thermometers were calibrated at the water triple point, 10 °C, 20 °C, 30 °C, 40 °C, and 50 °C [4]. In 1993, the thermometers were removed from the apparatus and recalibrated over an extended temperature range at −40 °C, 0 °C, 40 °C, 80 °C, and 120 °C. All temperatures in the 1993 calibration were based on the International Temperature Scale of 1990 (ITS-90). Based on the expanded uncertainty (*k* = 2) for the calibration bath temperatures of 0.01 K (Type B evaluation), the standard uncertainty was 0.005 K (*k* = 1). Recently, the cold plate PRTs have been removed from their respective plates and again submitted for calibration by the NIST Thermometry Group. These results will be updated when the most recent calibration and analysis are completed.

#### u (T_3_)—Other Small or Negligible Contributors

Several small or negligible contributory effects include the following: 1) PRT self heating/contact resistance; 2) sampling of temperatures in the meter area (*r*, θ); and, 3) temperature variations in the axial (*z*) direction (Fig. 5c). It is difficult to quantify the uncertainties of these contributors by separate experiments and, in some cases, the uncertainties are based on theoretical calculations or experimenter judgment. Hence, in all cases, the uncertainties are Type B evaluations.

1. *PRT self-heating/contact resistance*–The PRT excitation current is 1 mA which, for a nominal 100 Ω PRT, dissipates about 0.0001 W. For the meter plate PRT, a thin layer of thermally conductive silicone paste has been applied around the sensor to improve thermal contact (Fig. 5c). For the cold plate PRTs, the thermal conductance of the metal-to-air-to-metal interface between sensor and plate is estimated to be 0.058 W · K^−1^. Thus, the temperature rise (0.0001 W/0.058 W · K^−1^) is 0.0017 K.
2. *Sampling* (*planar*)—Rennex [4] and Siu [16] empirically determined the temperature profiles of different NIST meter plates utilizing independent thermopile constructions. In each experiment, the thermopiles were placed on the plate surfaces and a test conducted with semi-rigid specimens. Based on the thermopile measurements, Rennex [4] ascribed an estimate for the sampling uncertainty to be 0.015 K.
3. *Axial temperature variations*—A rigorous analytical analysis by B. A. Peavy published in Hahn et al. [12] shows that, for typical insulations, the differences between the temperature at the guard gap and the average surface temperature of the meter plate is less than 0.05 % of the temperature differences between the hot and cold plates. For a specimen temperature difference of 22.2 K, the standard uncertainty is 0.011 K.

#### u_c_(T)

Table 7 summarizes the sources, sensitivity coefficients (*c_i_*), uncertainty components *u*(*T_i_*), and the evaluation method (Type A or B) for the plate temperature. Application of Eq. (6) to the uncertainty components in Table 7 with *c_i_* = 1 yields a value for *u_c_*(*T*) of 0.061 K (Table 7, last row). For a Δ*T* of 22.2 K, *u_c,r_*(*T*) is 0.27 %. By comparison, C177-04 specifies an uncertainty for temperature sensors of less than 1 %. The dominant component for *u_c_*(*T*) in Table 7 is the uncertainty specification for the DMM measurement of the PRT electrical resistance.

#### uc (ΔT)

Recall from Eq. (10) that the specimen temperature difference (Δ*T*) was determined from the following equation:

(16) $$\Delta T=(T_{h}-T_{c}).$$

The application of Eq. (6) to Eq. (16) and setting $u_{T_{h}}=u_{T_{c}}=u_{T}$ yields

$$u_{c}(\Delta T)=\sqrt{u_{T_{h}}^{2}+u_{T_{c}}^{2}}=\sqrt{2u_{T}^{2}}.$$

Substitution of *u_c_* (*T*) = 0.061 K (Table 7) into Eq. (17) yields a value for *u_c_* (Δ*T*) of 0.086 K. For Δ*T* of 22.2 K, *u_c,r_* (Δ*T*) is 0.39 % (and for single-sided tests conducted (for customers) at a Δ*T* of 27.8 K, (Δ*T*) decreases to (0.31 %). Note that the value for *u_c_* (Δ*T*) of 0.086 K was used in the uncertainty assessment for the meter area (*A*).

### Heat Flow (Q)

Equation (10) defines the specimen heat flow (*Q*) as the difference between the power input to the meter plate (*Q_m_*) and parasitic heat losses (*Q_gap_*, *Q_ε_*, and *Q*′). Ideally, in the single-sided mode of operation (Fig. 2), the temperatures of the guard plate, ambient gas temperature, and auxiliary cold plate are maintained such that the parasitic heat losses are reduced to negligible proportions in comparison to *Q_m_*. Thus, *Q* is primarily determined by measuring the DC voltage and current provided to the meter-plate heater (*Q_m_*). The equation for *Q_m_* is:

$$Q_{m}=iV_{m}=\frac{V_{s}}{R_{s}}V_{m}$$

where *i* is the current (*V_s_*/*R_s_*) measured at the standard resistor, and *V_m_* is the voltage drop to the meter-plate heater measured across the voltage taps located at the midpoint of the guard gap.

The application of Eq. (6) to Eq. (18) yields

(19) $$u_{c}(Q_{m})=\sqrt{c_{V_{s}}^{2}u^{2}{\left(V_{s}\right)}^{2}+c_{R_{s}}^{2}u^{2}{\left(R_{s}\right)}^{2}+c_{V_{m}}^{2}u^{2}{\left(V_{m}\right)}^{2}}$$

with

$$\begin{array}{l} c_{V_{s}}=\partial (Q_{m})/\partial V_{s}=V_{m}/R_{s}\\ c_{R_{s}}=\partial (Q_{m})/\partial R_{s}=-\frac{V_{s}}{R_{s}^{2}}V_{m}\\ c_{V_{m}}=\partial (Q_{m})/\partial V_{m}=V_{s}/R_{s} \end{array}$$

#### u (Q_m_)—Power Input

The contributory sources *u*(*Q_m_*) for the meter-plate power input are discussed in detail below. Three contributory effects comprise *u*(*Q_m_*): 1) calibration of the standard resistor (Type B evaluation); 2) PRT self-heating (Type B evaluation); and 3) voltage measurements for *V_s_* and *V_m_* (Type A and Type B evaluations).

**Fig. 8:**
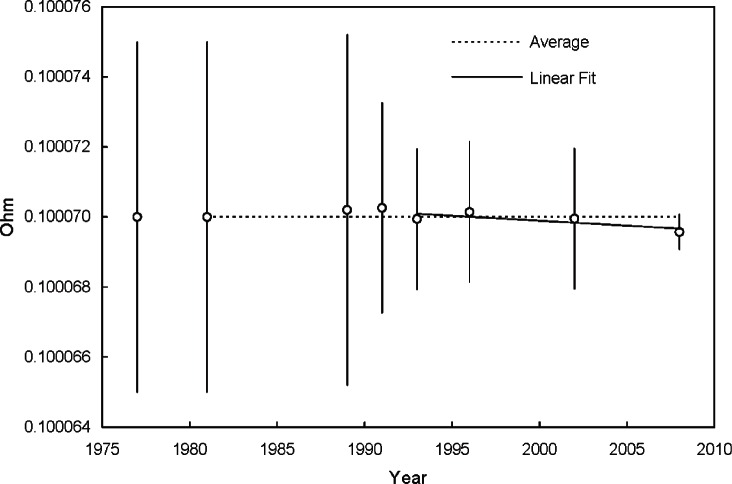
Control chart for 0.1 Ω standard resistor, S/N 21736

1. *Standard resistor calibration:* The 0.1 ohm standard resistor is a commercial, double-walled manganin resistor [17] manufactured in 1913. The resistor has been calibrated by the NIST Quantum Electrical Metrology Division in an oil bath at 25.00 °C for several years, most recently in 2008. Figure 8 shows the historical control chart for the resistor from 1977 to 2008. Since January 1, 1990, the NIST calibrations have been based on the quantum Hall effect used as the U.S. representation of the ohm [18]. The most recent calibration assigned the resistor a value of 0.10006957 Ω and an expanded uncertainty (*k* = 2) of 0.0000005 Ω. Therefore, the standard uncertainty was 0.00000025 Ω (*k* = 1).
  Careful inspection of Fig. 8 reveals a possible drift in the data and its presence could be indicative of other detrimental factors affecting the resistor itself. Annual calibrations are now planned to investigate the extent of the possible drift. During operation with guarded-hot-plate apparatus, the standard resistor is immersed in an oil bath maintained at 25.00 °C as shown in Fig. 4. Because the resistor is operated at the same temperature as the calibration temperature of 25.00 °C, temperature effects during operation were neglected.
2. *PRT power input:* As shown in Fig. 5c, the meter-plate PRT is fastened to the side of the meter plate. Under normal operating conditions, the PRT will generate a small power input to the meter plate due to self-heating effects. The excitation current for the meter-plate PRT is 1 mA which, for the nominal 100 Ω PRT, dissipates a power of about 0.0001 W. In the worst case for a 228.6 mm (9.0 in) thick specimen, the power input to the meter-plate heater is about 0.6 W and the PRT self-heating effect is 0.0001 W/0.6 W = 0.02 %. Thus, the effect of PRT self-heating was neglected for all specimen thicknesses.
3. *Voltage measurement:* Two contributory effects comprise the voltage measurement: 1) multiple observations (Type A evaluation); and, 2) the DMM voltage measurement uncertainty (Type B evaluation). During a typical CTS test (4 h in duration), *V_s_* and *V_m_* are recorded every minute by an automated data acquisition system (*n* = 240). The Type A uncertainty evaluations for *V_s_* and *V_m_* are included later (in Table 16) as repeated observations for the input power *u_A_*(*Q_m_*). The Type B standard uncertainty for the voltage measurements *V_s_* and *V_m_* (Fig. 4) assumes a uniform distribution with an interval 2*a* [2]; thus, $u_{B}=a/\surd \bar{3}$; where *a* was determined from the 1-year DMM specification. The DMM ranges for *V_s_* and *V_m_* are 30 mV and 30 V, respectively, and the corresponding values for *a*_30mV_ and *a*_30V_ are 15.0 μV and 3.05 mV at *L* = 25.4 mm. Therefore, *u_B_*(*V_s_*) and *u_B_*(*V_m_*) are 8.7 μV and 1.76 mV, respectively. Note that calibration checks for the DMM are conducted every other year; the last check was in 2008.

Table 8 summarizes the input estimates (*x_i_*), sensitivity coefficients (*c_i_*), and standard uncertainties (*u*(*x_i_*) for a CTS specimen thickness of 25.4 mm. Only Type B evaluation methods are included in Table 8. As stated above, the Type A uncertainty evaluations for *V_s_* and *V_m_* are included (in Table 16) as repeated observations for input power *u_A_*(*Q_m_*). Substituting the values in Table 8 into Eq. (19) yields a combined standard uncertainty for *Q_m_* of 0.0016 W or about 0.03 % for an input to the meter-plate heater of 5.15 W. The combined standard uncertainties at other specimen thicknesses are summarized later in this section.

*u*(*Q_gap_*), *u*(*Q*′), and *u*(*Q_ε_*)—*Parasitic Heat Flows:* Although the parasitic heat flows are reduced during steady-state conditions to very small values (on the order of 1 mW, or less), the uncertainty associated with each term can be large as shown below. The sources (*x_i_*) for the parasitic heat flows are discussed individually below and, later, their respective uncertainties are determined collectively as part of an imbalance study.

1. *Q_gap_*—*Guard gap heat flow:* The model for heat flow across the gap developed by Woodside and Wilson [19] is given in Eq. (20).
  (20) $$Q_{\mathrm{gap}}=(q_{o}+c\lambda )\Delta T_{\mathrm{gap}}$$
  where; *q_o_* represents the heat flow directly across the gap; *cλ* is the heat flow distortion in the insulation specimen adjoining the gap (Fig. 2); and Δ*T_gap_* is the temperature difference across the guard gap. Here, the term *λ* is the specimen thermal conductivity. The terms *q_o_* and *c* are a function of the apparatus design, specimen thickness, and thermal conductivity[19].
  Empirically, *Q_gap_* is determined from the thermopile voltage (*V_gap_*) of an eight junction (4 pairs) Type E thermopile across the guard gap.
  (21) $$Q_{\mathrm{gap}}^{\prime }=S_{\mathrm{gap}}(Sn\Delta T_{\mathrm{gap}})=S_{\mathrm{gap}}V_{\mathrm{gap}}$$
  where *S_gap_* is the heat flow sensitivity in W · μV^−1^, *S* is the Seebeck coefficient for Type E thermocouples in μV · K^−1^, and *n* is the number of junction pairs. At a meter plate temperature (*T_h_*) near 308 K (35 °C), the value of *S* is equal to 61.87 μV · K^−1^ [20]; thus, the sensitivity of the 8-junction (4 pair) thermopile is 4 × 61.87 μV · K^−1^ = 247.5 μV · K^−1^. For a DMM resolution of 0.1 μV, the temperature resolution of the thermopile is 0.0004 °C. Under balanced control, the variability of the gap thermopile voltage (*V_gap_*), determined from actual test data, is typically 1.5 μV or about 0.01 K (at a 3 × standard deviation level).
2. *Q*′—*Auxiliary insulation heat flow:* Equation (4) predicts the heat flow (*Q*′) through the meter section of the auxiliary insulation, which under normal one-sided operation is approximately zero. With the exception of *C*′, the quantities in the right-side of Eq. (4), namely *A*, *T*′*_h_*, and *T*′*_h_*, are determined from the measurement test data. One method for determining the thermal conductance (*C*′) of the auxiliary insulation (in W · m^−2^ · K^−1^) is an iterative technique described in Annex Al of Practice C1044 [11]. After the value of *C*′ is obtained, the standard uncertainty of *Q*′ can be determined by propagation of uncertainty in Eq. (4). An alternate method is to determine the value of the product of *C*′ *A* (that is, the thermal conductance per unit temperature (W · K^−1^)) from the imbalance study described below. In this case, the standard uncertainty is propagated through the mathematical model developed for the imbalance study.
3. *Q_ε_*—*Effect of edge heat transfer:* In general terms, the edge heat flow error is the distortion of one-dimensional heat flow through the specimen meter area due to heat flows at the periphery of the specimen. Edge effects are controlled by appropriate guarding in the design of the hot plate, limiting the specimen thickness, controlling the ambient temperature at the specimen edge, and, if necessary, the use of edge insulation. The empirical study by Orr [21] investigated the effects of edge insulation and changes in ambient temperature on edge heat flow error. A similar approach to determine the sensitivity of this error by varying the ambient air temperature for different specimen thicknesses was implemented as part of the imbalance study discussed later.

ASTM Practice C 1043 [5] provides a theoretical analysis of edge heat loss or gain based on analytical solutions derived by Peavy and Rennex [22] for both circular and square plate geometries. The purpose of the analysis is to provide the user of Practice C 1043 with design guidance in determining the proper diameter of the guard plate (Fig. 2) for control of edge heat loss or gain. An abbreviated version of the analysis is given below. The error (*ε*) due to edge heat transfer for either geometry is given by

(22) $$\varepsilon =A+BX\;\text{where}\;X=\frac{2(T_{m}-T_{a})}{T_{h}}-T_{c}$$

where *T_h_* and *T_c_* are the hot and cold plate temperatures, respectively (Fig. 2); *T_a_* is the ambient temperature at the edge of the specimen (Fig. 2); and, *T_m_* is the mean specimen temperature given by (*T_h_* + *T_c_*)/2. For a circular plate geometry, coefficients *A* and *B* are given by:

$$A=\sum_{n=1}^{\infty }W_{2n}\;\text{and}\;B=\sum_{n=1}^{\infty }W_{2n-1}$$

where

$$W_{n}=\frac{4}{\pi^{2}}\left(\frac{hL}{\lambda }\right)\left(\frac{\gamma L}{b}\right)\frac{I_{1}(n\pi b/\gamma L)}{n^{2}\left[I_{1}(n\pi d/\gamma L)+\frac{hL}{n\pi \lambda }I_{0}(n\pi d/\gamma L)\right]}.$$

The terms *I*_0_ and *I*_1_ are modified Bessel functions of the first kind of order 0 and 1, respectively. The term *b* is the radius of gap center; *d* is the radius of the hot plate; *L* is the specimen thickness; and, *h* is the convective film coefficient at the edge of the specimen. The term (*hL*/*λ*) is the Biot number; *γ* 2 = *λ_r_*/*λ_z_* where *λ_r_* and *λ_z_* are the radial and axial thermal conductivities of the specimen, respectively. The geometrical mean of the thermal conductivities is *λ* = (*λ_r_*·*λ_z_*)^1/2^.

The following results, for which the author is indebted to D. R. Flynn, retired from NIST, are presented in Table 9 for the following parameters:

$$\frac{hb}{\lambda }=40\;\text{and}\;X=\frac{2(T_{m}-T_{a})}{T_{h}-T_{c}}=\frac{2(5)}{22.2}=0.450.$$

Table 9 summarizes coefficients *A* and *B*, and the error (*ε*) due to edge heat transfer for specimen thicknesses of 25.4 mm, 76.2 mm, 152.4 mm, and 228.6 mm.

The error (*ε*) for *X* = 0 is essentially zero up to thicknesses of 228.6 mm. For an ambient temperature difference of ± 5 K from the mean specimen temperature (*X* = + 0.450), predicted values of *s* become significant at thickness of 152.4 mm.

#### ΔQ—Imbalance study

A series of imbalance tests were conducted to investigate empirically the effects of moderate temperature differences on *Q_gap_*, *Q*′, and *Q_ε_*. An imbalance test, as the name implies, is an operating condition in which a parameter (in this case, temperature difference) is purposely imbalanced (from zero) such that a parasitic heat flow is enhanced (i.e., the magnitude of the heat flow is either increased or decreased). In this study, imbalance tests are conducted for temperature differences across: 1) the guard gap (Δ*T_gap_* or, in this case, *V_gap_* as shown in Eq. (21)); 2) auxiliary insulation (*T*′*_h_* − *T*′*_c_*); and, 3) the mean specimen temperature at the specimen edge and the ambient air temperature (*T_m_* − *T_a_*).

**Fig. 9:**
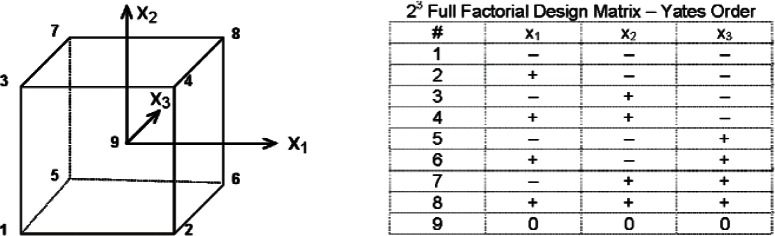
Full-factorial experimental design for 3 factors, 2 levels.

These parameters were varied following an orthogonal experimental design illustrated in Fig. 9, where *x*_1_ = *V_gap_*; *x*_2_ = *T*′*_h_* − *T*′*_c_*; and *x*_3_ = *T_m_* − *T_a_*. The test plan for the imbalance tests is based on a 2^3^ full factorial design meaning that the three factors are each varied at one of two levels shown in Yates order in the adjoining design matrix (with levels “coded” +1 for a high setting and −1 for a low setting). One advantage of an orthogonal design is that any interactions between factors (*x*_l_*x*_2_, *x*_1_*x*_3_, *x*_2_*x*_3_, and *x*_l_*x*_2_*x*_3_) can be detected; that is not possible for an experimental design that varies “one-factor-at-a-time.” Note that the experimental design given in Fig. 9 also contains a center point (#9), that is, a balanced test point where all imbalance settings have been set equal to zero.

For a 2^3^ full factorial design, the useful underlying mathematical model would be

(23) $$y=\beta_{0}+(\beta_{1}x_{1}+\beta_{2}x_{2}+\beta_{3}x_{3}+\beta_{12}x_{1}x_{2}+\beta_{13}x_{1}x_{3}+\beta_{23}x_{2}x_{3}+\beta_{123}x_{1}x_{2}x_{3})/2$$

where:

*x_i_* = “coded” level (+l or −1) for factor *i*;
*β_i_* = main effect for coded *x_i_*;
*β_ij_* = two-factor interaction effect for coded *x_i_*; and,
*β_ijk_* = three-factor interaction effect for coded *x_i_*.

Table 10 summarizes the input settings for the nine test conditions following the procedure given in Fig. 9. For tests #1 through #8, the “non-coded” settings for *x*_1_, *x*_2_, and *x*_3_ are ± 50 μV, ± 0.5 K, and ± 5 K, respectively. The input values for the test settings were selected with the objective of providing adequate responses in the parasitic heat flows. Test #9 is a balanced condition where each parameter is set to zero and the corresponding parasitic heat flows consequently approach zero. Note that Test #9 is an independent check test for the original test measurement.

The actual test sequence (not shown in Table 10) was randomized in order to minimize the introduction of bias in the results. The guarded-hot-plate imbalance tests were conducted with low-density fibrous-glass CTS specimens having thicknesses of 25.4 mm, 76.2 mm, 152.4 mm, and 228.6 mm (*n* = 36 data points) at a mean temperature of 297 K and a Δ*T* of 22.2 K across the specimen. The apparatus was operated in the single-sided mode of operation utilizing a specimen of expanded polystyrene foam having a nominal thickness of 100 mm as the auxiliary insulation (Fig. 2).

Table 11 summarizes the 36 values for *Q_m_*, Δ*T*, *V_gap_*, *T*′*_h_* − *T*′*_c_*; and, *T_m_* − *T_a_* at specimen thickness of 25.4 mm, 76.2 mm, 152.4 mm, and 228.6 mm. For a particular specimen thickness, values of *Q_m_* varied considerably, depending on the imbalance settings for *x*_l_ = *V_gap_*; *x*_2_ = *T*′*_h_* − *T*′*_c_* ; and, *x*_3_ = *T_m_* − *T_a_*. The balanced condition provided in Test #9 was used to establish a baseline value for analyses of the data. At the balanced condition (Test #9), values of *Q_m_* decreased from about 5.1 W to 0.6 W as the specimen thickness increased from 25.4 mm to 228.6 mm. This 8 fold decrease in *Q_m_* is important and will be treated later.

Values of *Q_m_* from Table 11 and coded values (±1) of *x*_1_, *x*_2_, and *x*_3_ were input into Eq. (23) and the least-squares estimated effects (*β_i_*, *β_ij_*, and *β_ijk_*) were calculated using a NIST statistical graphical analysis program [23] that employed Yates algorithm [24]. Table 12 summarizes whether the estimated effects (*β_i_*, *β_ij_*, and *β_ijk_*) are statistically significant at the 5 % level or the 1 % level. In all cases, factors *x*_1_ and *x*_2_ were significant. Somewhat surprisingly, however, factor *x*_3_ was determined to be insignificant for *L* = 152.4 mm and 228.6 mm. The effects estimates for all interactions (*x*_1_*x*_2_, *x*_1_*x*_3_, *x*_2_*x*_3_, and *x*_1_*x*_2_*x*_3_) across all thicknesses were insignificant. Based on these results, the (non-coded) data in Table 11 were subsequently fitted to a simplified model (discussed below).

The parasitic heat flows from the meter area, expressed mathematically in Eq. (10), represent lateral heat flows due to temperature differences across the guard gap and the auxiliary insulation, and due to specimen edge effects. Based on the results from Table 12, the data from Table 11 were fit to an empirical model for Δ*Q* given in Eq. (24) below,

(24) $$\Delta Q=Q_{m_{i}}-Q_{m_{0}}=a_{1}V_{\mathrm{gap}}+a_{2}(T_{h}^{\prime }-T_{c}^{\prime })+a_{3}(T_{m}-T_{a})$$

where:

*Q_mi_* = the power input to the meter-plate heater for test *i* = 1 to 9;
$Q_{m_{0}}$ = the power input to the meter-plate heater for test *i* = #9 (balanced condition);
*a_j_* = regression coefficients (*j* = 1,2,3);
*V_gap_* = guard gap voltage corresponding to Δ*T_gap_* (μV);
*T*′*_h_* = hot plate temperature (K);
*T*′*_c_* = auxiliary plate temperature (K);
*T_m_* = mean specimen temperature (K) = (*T_h_* + *T_c_*)/2; and,
*T_a_* = ambient gas temperature near the edge of the specimen (K).

The presence of an offset coefficient *a*_0_ was considered for the model given in Eq. (24) but, because the term is predicted to be nearly zero from theory (Eq. 22), the term is not included here.

Table 13 summarizes estimates and approximate standard deviations determined by multiple variable linear regression for coefficients, *a*_l_, *a*_2_, and *a*_3_ as a function of specimen thickness. The “goodness of fit” denoted by the residual standard deviation (RSD) in the last column is equal to, or less than, 3 mW (Type A evaluation). If an offset coefficient *a*_0_ is incorporated in Eq. (24), the RSDs for the fits are about 1 mW, or less. Regression coefficients *a*_l_, *a*_2_, and *a*_3_ are discussed below.

1. *a*_1_: Estimates for *a*_l_ in Table 13 represent the heat flow sensitivities (*S_gap_*) in W·μV^−1^ for the 4-pair thermopile as defined in Eq. (21). At 25.4 mm, the *a*_l_ estimate is consistent with previous results^8^ obtained for SRM 1450c, Fibrous Glass Board [3]. Note, however, that estimates fo *a*_l_ increase 8.2 % from 25.4 mm to 228.6 mm indicating that, for a fixed temperature difference, the heat-flow across the guard gap increases with the specimen thermal resistance. From Eq. (20) and Table 1, the ratio of gap heat flow and specimen heat flow is
  $$\frac{Q_{\mathrm{gap}}}{Q}=\frac{(q_{o}+c\lambda )\Delta T_{\mathrm{gap}}}{A\Delta T/R}$$
  or
  (25) $$\frac{Q_{\mathrm{gap}}}{Q}=\frac{1}{A}\frac{\Delta T_{\mathrm{gap}}}{\Delta T}(q_{o}R+cL).$$
  Therefore, for a particular apparatus (i.e., fixed *A*) and a given temperature imbalance (Δ*T_gap_*), the ratio of lateral and specimen heat flows increases as the specimen resistance (*R*) and thickness (*L*) increase if the other factors (namely Δ*T*) remain unchanged. Consequently, if one assumes a constant coefficient for *a*_l_ based on *L* equal to 25.4 mm, an error in uncertainty is introduced for tests conducted at greater thicknesses.
2. *a*_2_: Estimates for *a*_2_ in Table 13 represent the thermal conductance per unit temperature (C*′_a_*) of the auxiliary specimen (100 mm thick polystyrene foam) at 308 K (35 °C). The average value for Table 13 is −0.04843 W·K^−1^ and range is 0.00022 W·K^−1^ (or 0.5 %). As a check, the values for *a*_1_ were compared with computed values obtained for the thermal conductance (C*′_a_*) computed from Eq. (26) below:
  (26) $$C_{a}^{\prime }=C^{\prime }A=\frac{k^{\prime }A}{L^{\prime }}.$$
  Substitution of *k′* = 0.0373 W·m^−1^·K^−1^ obtained from independent guarded-hot-plate tests at 311 K, *A* = 0.12989 m^2^ at 308 K, and *L′* = 0.1013 m in Eq. (26) yields a value of 0.0478 W·K^−1^ which is within 1.3 % of ${\bar{a}}_{2}$.
3. *a*_3_: Although the estimates for *a*_3_ in Table 13 represent a thermal conductance per unit temperature, it is more useful to express these estimates as an edge heat flow error (*ε*). By setting *V_gap_* = 0, (*T′_h_* − *T′_c_*) = 0, and dividing by $Q_{m_{0}}$, Eq. (24) becomes
  (27) $$\frac{\Delta Q}{Q_{m_{0}}}=\frac{Q_{m_{i}}-Q_{m_{0}}}{Q_{m_{0}}}=\frac{a_{3}}{Q_{m_{0}}}(T_{m}-T_{a}).$$
  Table 14 summarizes values of *ε* computed from Eq. (27) for (*T_m_* − *T_a_*) equal to 0 K and ± 5 K and values of *ε* reproduced from Table 9 for *L* ranging from 25.4 mm to 228.6 mm. Theoretical values of *ε* are based on calculations from Eq. (22) for the same values of temperature imbalances. There are two general conclusions from the results presented in Table 14:
  - At specimen thicknesses less than or equal to 76.2 mm, the measured effect is small but greater than predicted by theoretical analysis. The fact that the empirical results are not monotonic with *L* suggests that the observed variations for 152.4 mm and below are due to experimental variations of factors other than *L*.
  - At specimen thicknesses greater than or equal to 152.4 mm, the measured effect is much smaller than predicted by theoretical analysis.

Further work is recommended to investigate the differences between edge heat flow errors computed from empirically-derived models and from theoretical based models.

#### u_c_(ΔQ)—Standard uncertainty for parasitic heat flows

Substitution of *x*_1_ = *V_gap_* ; *x*_2_ = *T′_h_* − *T′_c_* ; and, *x*_3_ = *T_m_* − *T_a_* for the quantities in Eq. (24) and application of Eq. (6) yields:

(28) $$u_{c}(\Delta Q)=\sqrt{\begin{array}{l} c_{a_{1}}^{2}u^{2}(a_{1})+c_{x_{1}}^{2}u^{2}(x_{1})+c_{a2}^{2}u^{2}(a_{2})\\ +c_{x_{2}}^{2}u^{2}(x_{2})+c_{a_{3}}^{2}u^{2}(a_{3})+c_{x3}^{2}u^{2}(x_{3}) \end{array}}$$

with

$$\begin{array}{l} c_{a_{j}}=\partial (\Delta Q)/\partial a_{j}=x_{j}\\ c_{x_{j}}=\partial (\Delta Q)/\partial x_{j}=a_{j}. \end{array}$$

Table 15 summarizes input values for Eq. (28) and the corresponding *u_c_* (Δ*Q*) for specimen thicknesses of 25.4 mm, 76.2 mm, 152.4 mm, and 228.6 mm. The input values for *u* (*a_i_*) and *a_i_* were obtained from Table 13. Under steady-state test conditions, the input estimates for *x_i_* are nearly zero (Table 15). The standard uncertainties for *x*_1_ (Δ*T_gap_*), *x*_2_ (*T′_h_* − *T′_c_*), and *x*_3_ (*T_m_* − *T_a_*) were estimated to be ± 0.01 K, which corresponds to ± 2.48 μV for *V_gap_*, ± 0.086 K and ± 0.5 K, respectively. Note that the resulting values of *u_c_* (Δ*Q*) are nearly constant across all levels of specimen thickness.

#### u_c_ (Q)—Standard uncertainty for specimen heat flow

Recalling the heat balance for the meter area given in Eqs. (10) and (11), the specimen heat flow (*Q*) was determined from the following equation:

(29) $$Q=Q_{m}-\Delta Q.$$

Application of Eqs. (6) and (7) to Eq. (29) yields

(30) $$u_{c}Q=\sqrt{u_{A}^{2}(Q_{m})+u_{B}^{2}(Q_{m})+u_{c}^{2}(\Delta Q)}$$

where the sensitivity coefficients are equal to unity and the contributory uncertainties are

*u_A_* (*Q_m_*) = standard uncertainty of repeated power measurements (*n* = 240) during a test (W);
*u_B_* (*Q_m_*) = standard uncertainty of the meter-plate power input—see Table 8 (W); and,
*u_c_* (Δ*Q*) = combined standard uncertainty for parasitic heat flows computed in Eq. (28)—see Table 15 (W).

Table 16 summarizes the estimates for the combined standard uncertainty *u_c_* (*Q*) across all thicknesses. The dominant source by, in many cases, two orders of magnitude is the uncertainty in the parasitic heat flows which, in turn, is due primarily to the uncertainty in the gap thermopile voltage and temperature difference across the auxiliary insulation. The relative combined standard uncertainty *u_c,r_* (*Q*) increases considerably with specimen thickness from 0.17 % at 25.4 mm to 1.4 % at 228.6 mm.

## 10. Calculation of Combined Uncertainty

The standard uncertainties for meter area (*A*). thickness *(L*). temperature difference (Δ*T*), and power (*Q*) calculated in the previous section are combined using the law of propagation uncertainty given in the GUM (Eq. (6)). The combined standard uncertainties (*u_c_*, *u_c,r_*) for *λ_exp_*, and *R* are presented for specimens of low-density fibrous-glass thermal insulation at thicknesses of 25.4 mm, 76.2 mm, 152.4 mm, and 228.6 mm. The expanded uncertainties (*U*, *U_r_*) for *λ_exp_* and *R* are also presented for the same thicknesses using a coverage factor of *k* = 2.

### Combined Standard Uncertainty and Expanded Uncertainty for λ_exp_

Application of Eq. (6) to Eq. (10) yields the combined standard uncertainty for *λ_exp_*:

(31) $$u_{c}(\lambda_{\exp})=\sqrt{c_{Q}^{2}u_{c}^{2}(Q)+c_{L}^{2}u_{c}^{2}(A)+c_{\Delta T}^{2}u_{c}^{2}(\Delta T)}$$

with

$$\begin{array}{lll} c_{Q} & =\partial \lambda_{\exp}/\partial Q & =\frac{L}{A(\Delta T)}\\ c_{L} & =\partial \lambda_{\exp}/\partial L & =\frac{Q}{A(\Delta T)}\\ c_{A} & =\partial \lambda_{\exp}/\partial A & =\frac{-QL}{A^{2}(\Delta L)}\\ c_{\Delta T} & =\partial \lambda_{\exp}/\partial \Delta T & =\frac{-QL}{A{\left(\Delta T\right)}^{2}} \end{array}$$

Table 17 summarizes the input estimates (*x_i_*), sensitivity coefficients (*c_i_*), standard uncertainties (*u_c_* (*x_i_*)) for *Q*, *L*, *A*, and Δ*T* at thicknesses of 25.4 mm, 76.2 mm, 152.4 mm, and 228.6 mm. The last two columns of each table provide the absolute and relative contributions of each component uncertainty. The last two rows of each table provide *λ*_exp_, *u_c_*(*λ*_exp_), *u_c,r_*(*λ*_exp_), *U*, and *U_r_* using a coverage factor of *k* = 2. The estimate for *λ*_exp_ and corresponding bulk density (*ρ*) are also given on the last row of each table.

**Fig. 10:**
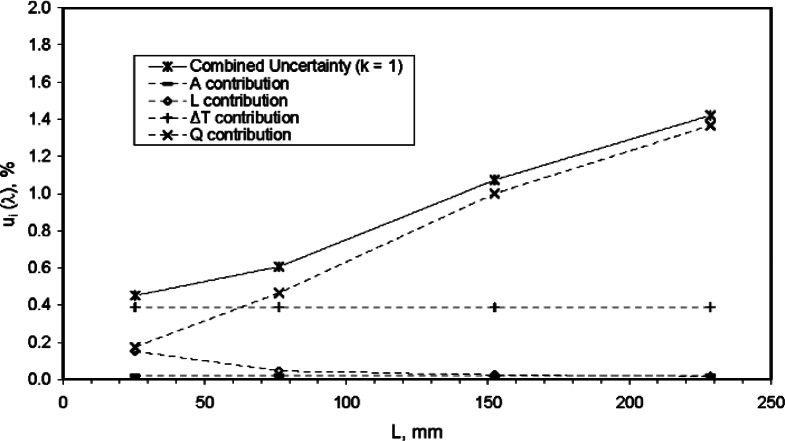
Combined standard uncertainty and individual components for *λ*.

Using the values summarized in Table 17, Fig. 10 plots the relative combined standard uncertainty for *λ* and individual uncertainty components for *Q*, *L*, *A*, and Δ*T* as a function of *L*. The graphical analysis clearly shows that the uncertainty contribution from *Q* is the major component of uncertainty, especially at thicknesses of 75 mm and above. At 25 mm, the major component is the standard uncertainty of Δ*T* which is fixed at 0.39 % across all levels of *L*. The uncertainty contribution due to *L* is largest at 25 mm and declines dramatically, as expected, as *L* increases. The uncertainty contribution due to *A* is nearly zero for all thicknesses.

**Fig. 11:**
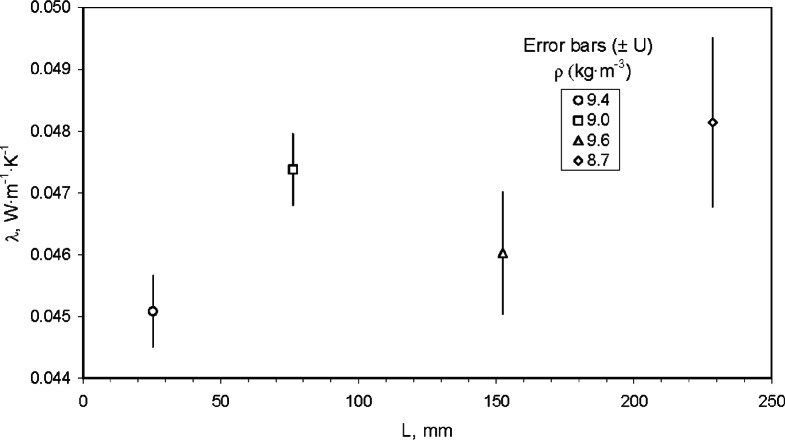
Thermal conductivity measurements of Fibrous-Glass Blanket NIST CTS as a function of thickness.

Figure 11 plots the estimates for thermal conductivity (*λ*) of the fibrous-glass blanket CTS given in Table 17 as a function of specimen thickness (*L*). The error bars shown in Fig. 11 are equal to the expanded uncertainties *U* (*k* = 2) given in Table 17. The data in Fig. 11 show a “thickness effect” which was the basis for the R-value Rule [9], and the subsequent development of the NIST CTS lot of material. The data in Fig. 11 also reveal a material variability factor for the NIST CTS lot of material.

### Combined Standard Uncertainty and Expanded Uncertainty for R

Application of Eq. (6) to Eq. (11) yields the combined standard uncertainty for *R*:

(32) $$u_{c}(R)=\sqrt{c_{Q}^{2}u_{c}^{2}(Q)+c_{A}^{2}u_{c}^{2}(A)+c_{\Delta T}^{2}u_{c}^{2}(\Delta T)}$$

with

$$\begin{array}{lll} c_{Q} & =\partial R/\partial Q & =\frac{A^{2}(\Delta T)}{Q^{2}}\\ c_{A} & =\partial R/\partial A & =\frac{\Delta T}{Q}\\ c_{\Delta T} & =\partial R/\partial \Delta T & =\frac{A}{Q}. \end{array}$$

Table 18 summarizes the input estimates (*x_i_*), sensitivity coefficients (*c_i_*), standard uncertainties (*u_c_* (*x_i_*)) for *Q*, *A*, and Δ*T* at thicknesses of 25.4 mm, 76.2 mm, 152.4 mm, and 228.6 mm. The last two columns of each table provide the absolute and relative contributions of each component uncertainty. The last two rows of each table provide *R*, *u_c_* (*R*), *u_c,r_* (*R*), *U* and *U_r_* using a coverage factor of *k* = 2.

**Fig. 12:**
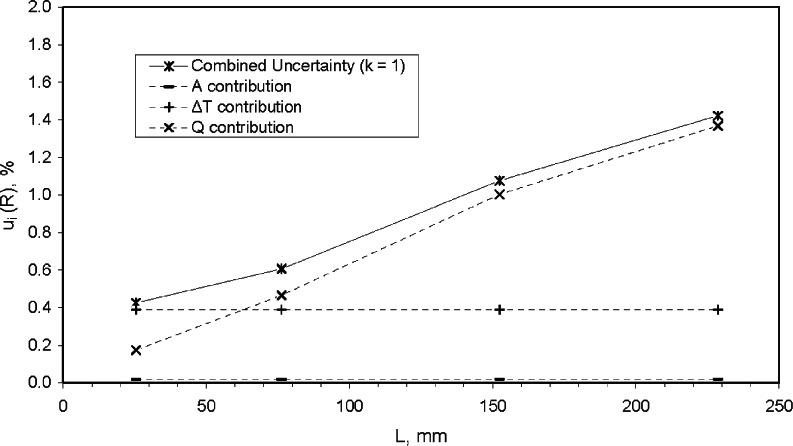
Combined standard uncertainty and individual components for R.

Using the values summarized in Table 18, Fig. 12 plots the relative combined standard uncertainty for *λ* and individual uncertainty components for *Q*, *A*, and Δ*T* as a function of *L*. The graphical analysis is very similar to Fig. 10 except for the absence of any uncertainty contribution from *L* in Fig. 12. The analysis again clearly shows that the contribution from *Q* is the major component of uncertainty, especially at thicknesses of 75 mm and above. The major component is the standard uncertainty of Δ*T* which is fixed at 0.39 % across all levels of *L*. The uncertainty contribution due to *A* is nearly zero for all thicknesses.

## 11. Reporting Uncertainty

The measurement result issued to a customer for a CTS specimen is for thermal resistance (*R*), not for thermal conductivity (*λ*_exp_). Therefore, the expanded uncertainty *U* and relative expanded uncertainty *U_r_* for thermal resistance (*R*) are reported as follows:

*Thermal Resistance* (*R*): *R*, m^2^·K·W^−1^ ± *U*, m^2^·K·W^−1^(*U_r_*, %)

where the reported uncertainty is on expanded uncertainty as defined in the International Vocabulary of Basic and General terms in metrology, 2nd ed., ISO 1993 calculated using a coverage factor (k) of 2. The results given in this report apply only to the specimen tested, and not to any other specimen from the same or from a different lot of material.

The relative expanded uncertainty *U_r_* for *R* is provided to a customer with two significant digits that are rounded up to the nearest 0.5 %. For example, the values of *U_r_* for fibrous-glass blanket NIST CTS given in Table 18 are rounded as shown in Table 19. Values of *U* for the customer are rounded to be consistent with *U_r_*

It is important to emphasize that other low-density fibrous-glass insulation materials may (and probably will) have different values of *R* and, consequently, different values of *U_r_* than those given in Table 19. Furthermore, it usually inappropriate to include in the uncertainty for a NIST result any component that arises from a NIST assessment of how the result might be employed [1]. These uncertainties may include, for example, effects arising from transportation of the measurement artifact to the customer laboratory including mechanical damage; passage of time; and differences between the environmental conditions at the customer laboratory and at NIST [1].

## 12. Discussion

**Fig. 13:**
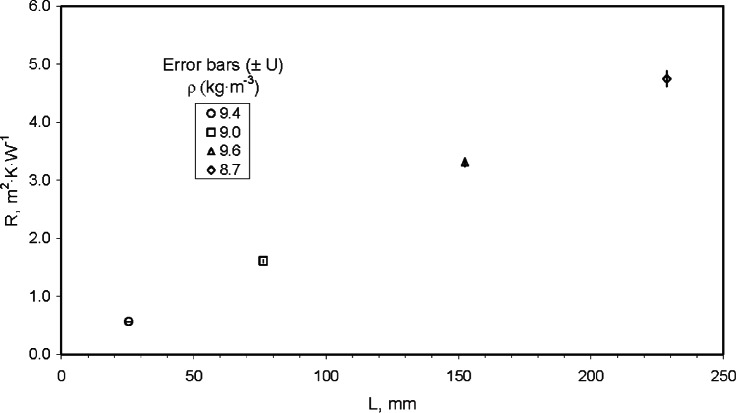
Thermal resistance measurements of Fibrous-Glass Blanket NIST CTS *(T_m_* of 297 K, Δ*T* of 22.2 K).

NIST issues low-density fibrous-glass blanket CTS taken from an internal lot of insulation at nominal thicknesses of 25 mm, 75 mm, 150 mm, or 225 mm. In general, measurements for customers are usually conducted at a mean temperature of 297 K and a temperature difference of either 22.2 K or 27.8 K. Figure 13 plots the nominal thermal resistance (*R*) of the reference material as a function of specimen thickness (*L*) at *T_m_* of 297 K and Δ*T* of 22.2 K. The error bars shown in Fig. 13 are equal to ±*U*(*k* = 2) given in Table 18.

**Fig. 14:**
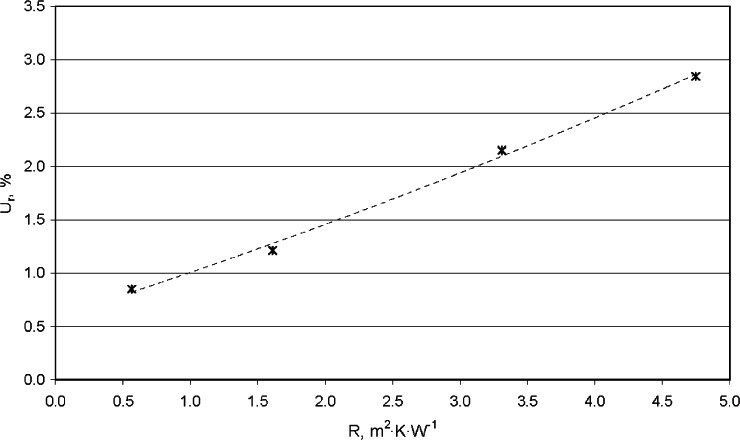
Relative expanded uncertainties (*k* = 2) versus thermal resistance of Fibrous-Glass Blanket NIST CTS.

Figure 14 examines the expanded uncertainties shown in Fig. 13 in greater detail by plotting *U_r_* from Table 18 as a function of *R*. It is interesting to note that the trend for the expanded uncertainty data is non-linear. There are two possible related explanations for this non-linear behavior. At high levels of *R*, the specimen heat flow is reduced considerably thereby increasing the sensitivity coefficients (*c_i_*) and *u_c_*(*Q*) as shown in Eq. (32). Also, high levels of *R* are generally due to thick insulation specimens which will increase the edge heat flow effects.

### Dominant Uncertainty Components

Table 20 summarizes the individual contributions in percent (%) at the *k* = 1 level for *A*, *L*, Δ*T*, and *Q* (presented in Tables 17 and 18) for specimen thicknesses of 25.4 mm, 76.2 mm, 152.4 mm, and 228.6 mm. At 25.4 mm, the dominant uncertainty component is for Δ*T* and, at thicknesses greater than 25.4 mm, the dominant component is for *Q* which increases considerably with *L*. The contribution of *u*(Δ*T*) is fixed for the measurements presented herein at 22.2 K; however, the contribution of *u*(Δ*T*) would change for different values of Δ*T*. It is interesting to note that the contribution of *L* on *λ*_exp_ is the largest at 25.4 mm, decreasing at higher specimen thicknesses. Conversely, one would expect that, based on the results in Table 20, *u**_c,r_* (*L*) would increase for specimen thicknesses less than 25.4 mm. In that case, *u_c_*(*λ*_exp_) could be larger than *u_c_*(*R*) because, strictly speaking, *u_c_*(*L*) does not directly affect the uncertainty computation for *R* as shown in Eq. (11). Finally, the small contribution for *A*, which is fixed for the all thicknesses, could change considerably at temperatures further from ambient due to thermal expansion effects (as noted above).

### Dominant Contributory Sources

To investigate the results presented in Table 20 further, it is useful to re-examine the contributory sources for each component uncertainty of *A*, *L*, Δ*T*, and *Q*. Figure 13 reproduces Fig. 7 with the dominant contributory source for each component identified (by circle). It is important to note that these dominant contributory sources may be different for other measurements (i.e., different specimen materials, operation temperatures, etc.). Furthermore, operators having other guarded-hot-plates will note that the dominant contributory sources given here do not necessarily apply to their own measurement process.

The contributory sources identified in Fig. 13 are discussed further below.

- *Meter area* (*A*): At *T_m_* of 297 K and Δ*T* of 22.2 K, *u_c,r_* (*A*) is fixed at an estimate of 0.02 % for all measurements presented in this report. The dominant contributory sources are the uncertainties in plate dimensions of the guard gap (Table 3). However, at temperatures departing from ambient, the contributory uncertainties for thermal expansion are expected to increase appreciably.
- *Specimen thickness* (*L*): The dominant contributory source for *L* is due to the uncertainty from the cold plate deflection under mechanical load (Table 6). This source is significant for compressible materials, such as thermal insulation blankets, and is typically smaller for more rigid and semi-rigid materials. In the case of rigid and semi-rigid materials, other contributory sources in Table 6 could become more important.
- *Temperature difference* (Δ*T*): The dominant contributory source for Δ*T* is the digital multimeter (DMM) measurement of the PRT temperature sensor which has a standard uncertainty of 0.058 K (Table 7). The standard uncertainty is based on the manufacturer specification and is probably a conservative estimate.
- *Heat flow* (*Q*): The dominant contributory source of *Q* is the uncertainty in the parasitic heat flows (Δ*Q*) across the guard gap and auxiliary insulation. The contributory sources for these parasitic heat flows are the control variability of the gap thermopile voltage of 1.5 μV or about 0.01 K (at the 3 standard deviation level) and the measurement of the PRT temperature sensors.

### Comparison With Previous Error Analysis

Officially, the uncertainty assessment presented herein supersedes the previous error analysis prepared in 1983 [4]. This means that only the uncertainty values presented in this report should be used for the NIST Calibrated Transfer Specimens. Nevertheless, there is technical merit in discussing both analyses. Some of the obvious differences are the changes in measurement processes from 1983 to 2008 that involve different operators as well as modifications to some of the equipment (including both the apparatus and the data acquisition system). There is also a fundamental difference in the two approaches taken for the determination of combined standard uncertainty as discussed below. An examination of both approaches also reveals similarities in how individual and contributory uncertainties were determined. A brief discussion of the differences and similarities of the two analyses is presented below.

#### Differences in approach

First and foremost, the error analysis prepared in 1983 [4] (hereafter, 1983 error analysis) preceded the official NIST policy [1] that adopted current international guidelines for the expression of measurement uncertainty [2]. Consequently, the terminology given in the 1983 error analysis, as well as the approach for combining the uncertainties, has been rendered out of date. Other specific differences in the two approaches are given below. The 1983 error analysis:

1. did not categorize the uncertainty components as either random or systematic as was done in an uncertainty analysis of an earlier NBS guarded-hot-plate apparatus [16]; and,
2. estimated an “upper bound” of the “total uncertainty” by direct summation of the uncertainty components.

In contrast, the GUM approach calculates a combined standard uncertainty by the root-sum-of-squares approach shown in Eq. (6). Further, the GUM and NIST policy [1] require that the expanded uncertainty (*U*) be reported with a coverage factor (*k*) equal to 2 for international comparisons. The 1983 error analysis does not report a coverage factor (*k*) and the “direct summation approach” makes it difficult to assess the coverage factor. Without a coverage factor (or a method to determine one) it is difficult, if not impossible, to compare the combined standard uncertainties from the two analyses.

#### Similarities in individual uncertainty components

A brief review of the individual uncertainties in the 1983 error analysis revealed that several of the estimates agree reasonably well with the values presented in Table 17 for the combined standard uncertainty of *λ*_exp_. Table 21 summarizes a side-by-side comparison of the individual uncertainties determined for this assessment and for the 1983 error analysis.

For *A* and *L*, the standard uncertainties determined in this assessment and estimates of uncertainties for the 1983 error analysis are in very good agreement. For Δ*T*, the standard uncertainties for this assessment are more conservative by a factor of approximately 2. As revealed in Table 21, the major differences in the individual components are the estimates for the specimen heat flow *Q*. The difference is attributed to the approach taken in this report for empirically determining the uncertainty in parasitic heat flows of the meter area. The resulting Type B standard uncertainty *u_B_*(Δ*Q*), as observed in Table 16, dwarfs the other contributions for heat flow (*Q*) by, in some cases, two orders of magnitude. The author believes that the approach presented here for determining estimates of *u_c_*(Δ*Q*) using an experimental imbalance study is necessary to determine the standard uncertainty for the specimen heat flow (*Q*).

## 13. Summary

An assessment of uncertainties for the National Institute of Standards and Technology (NIST) 1016 mm Guarded-Hot-Plate apparatus is described in this report. The uncertainties have been reported in a format consistent with current NIST policy on the expression of measurement uncertainty, which is based on recommendations by the Comité International des Poids et Mesures (CIPM) given in the *Guide to the Expression of Uncertainty in Measurement*. Strictly speaking, these uncertainties have been developed for a particular lot of low-density fibrous-glass blanket thermal insulation having a nominal bulk density of 9.6 kg · m^−3^. This reference material, known as a NIST Calibrated Transfer Specimen (CTS), is issued to customers as specimens 610 mm square and at nominal thicknesses of 25.4 mm, 76.2 mm, 152.4 mm, and now 228.6 mm for use in conjunction with the “representative thickness” provision of the U.S. Federal Trade Commission (FTC) R-value Rule. The relative expanded uncertainty at a coverage factor of *k* equal to 2 for the thermal resistance of this material increases from 1 % for a thickness of 25.4 mm to 3 % for a thickness of 228.6 mm. The approach for the assessment of uncertainties that has been developed herein is applicable to other insulation materials measured at 297 K.

### Recommendations for Future Research

The uncertainty analyses given in this report have identified dominant components of uncertainty and, thus, potential areas for future measurement improvement. For the NIST 1016 mm Guarded-Hot-Plate apparatus considerable improvement, especially at higher levels of *R*, may be realized by developing better control strategies for the guard gap that include better measurement techniques for the gap voltage and PRT temperature sensors. In some cases, determining the individual standard uncertainties has required establishing traceability to NIST metrology laboratories, specifically for thermometry and primary electrical measurements. Recent calibrations from these metrology laboratories have indicated that more frequent checks and/or calibrations are required in the future. An extensive list of recommendations and future activities is given below:

- *Annual check of platinum resistance thermometers (PRTs)*: The last calibration by the NIST Thermometry Group revealed a stability problem with one of the cold-plate PRTs. To track any drift or potential problem, an annual check of the cold-plate PRTs at the triple point of water is now planned.
- *Annual calibration of standard resistor*: The last calibration by the NIST Quantum Electrical Metrology Division revealed a possible drift in the standard resistor used for the meter-plate power measurement. The presence of the drift could be indicative of detrimental factors affecting the resistor itself. Annual calibrations are now planned to investigate the extent of the possible drift.
- *Improvement in thickness measurement*: The dominant component for the thickness uncertainty is the analysis of the plate deflection under mechanical load. Because the approach presented here has limitations, an alternative technique to assess the plate deflection more accurately could reduce the thickness uncertainty. This would be useful at specimen thicknesses of 25.4 mm or less.
- *Improvement in temperature measurement*: The dominant contributory source for the specimen Δ*T* is the digital multimeter (DMM) measurement of the PRT temperature sensor, which has a standard uncertainty of 0.058 K. The standard uncertainty for the DMM measurement is based on the manufacturer specification and is probably conservative. Further, the uncertainty probably includes systematic effects of unknown magnitude which will largely cancel when the specimen difference is computed in Eq. (16). A significant improvement in the temperature measurement could be realized by development of new instrumentation for measuring the PRT temperature sensors. This improvement would also result in a lower uncertainty for the parasitic heat flows as discussed below.
- *Reduction in the uncertainty of parasitic heat flows*: The uncertainties of the parasitic heat flows are due primarily to the uncertainty in temperature measurement (and control) of the guard gap thermopile and the temperature difference of the auxiliary plate temperatures. As discussed above, reduction in the temperature measurement uncertainty for PRTs would significantly reduce the uncertainty in the parasitic heat flow, which is the dominant component of the specimen heat-flow uncertainty.
- *Analysis of error due to edge heat transfer*: Further work is recommended to investigate the differences between edge-heat flow errors computed from theory and those computed from an empirically-derived model presented in this report.

## Figures and Tables

**Fig. 5a:**
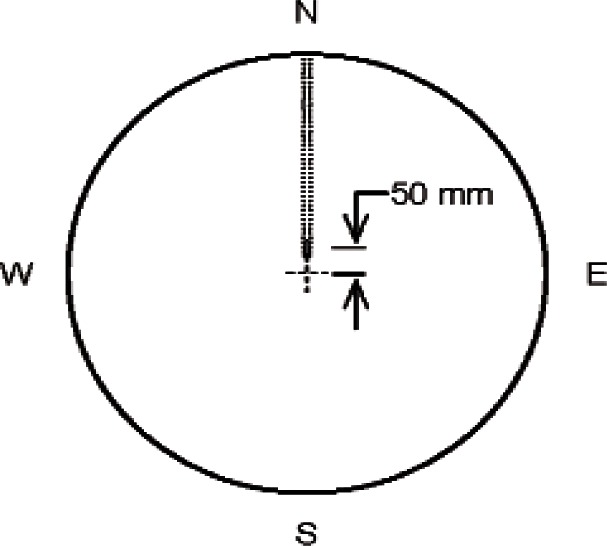
Location of cold plate PRT (top view).

**Fig. 5b:**
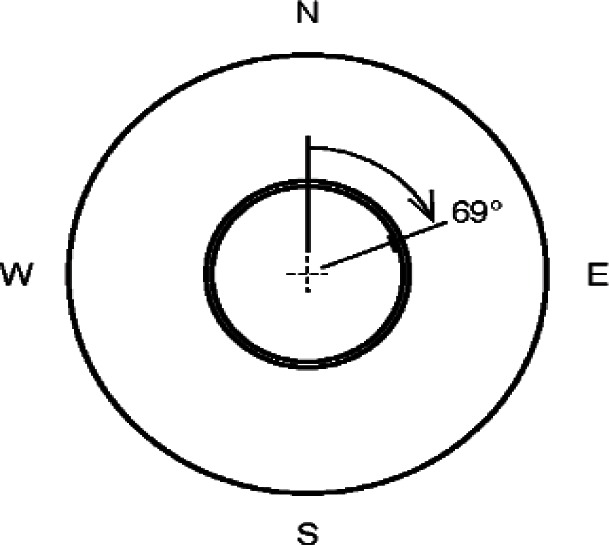
Location of hot plate PRT in guard gap on meter side of guard gap (top view).

**Fig. 5c:**
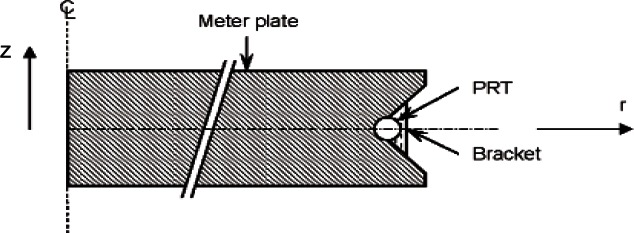
Cross-section view of PRT in guard gap (guard plate not shown).

**Fig. 15:**
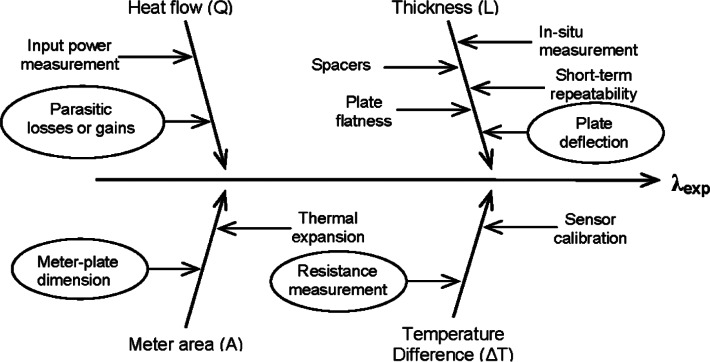
Cause-and-effect chart for *λ*_exp_ with dominant contributary sources identified.

**Table 1:** Steady-State One-Dimensional Thermal Transmission Property Equations

	Thermal Resistance *R*, m^2^·K·W^−1^	Thermal Conductance *C*, W·m^−2^·K^−1^	Thermal Resistivity *r*, m·K·W^−1^	Thermal Conductivity *λ*, W·m^−1^·K^−1^
Equation	$R=\frac{A\Delta T}{Q}$	$C=\frac{Q}{A\Delta T}$	$r=\frac{A\Delta T}{QL}$	$\lambda =\frac{QL}{A\Delta T}$
Relationships	$R=\frac{1}{C}=\frac{L}{\lambda }$	$C=\frac{1}{R}=\frac{\lambda }{L}$	$r=\frac{1}{\lambda }$	$\lambda =\frac{1}{r}$

**Table 2:** List of Uncertainty Sources for *λ* for the N1ST 1016 mm Guarded-Hot-Plate Apparatus

1) Meter area (*A*) Plate dimensions Thermal expansion effects
2) Thickness (*L*) In-situ linear position measurement system Multiple observations System uncertainty Dimensions of fused-quartz spacers Repeated observations Caliper uncertainty Short-term repeatability Plate flatness Repeated observations Coordinate measuring machine (CMM) uncertainty Plate deflection under axial loading of cold plate
3) Temperature difference (Δ*T*) Measurement (*T_h_*, *T_c_*) Digital multimeter (DMM) uncertainty PRT regression uncertainty in fit for calibration data Calibration of PRTs Miscellaneous sources (not shown in Fig. 7) Contact resistance Sampling of planar plate temperature Axial temperature variations
4) Heat flow (*Q*) DC power measurement (*Q_m_*) Standard resistor calibration Standard resistor drift PRT power input Voltage measurement Parasitic heat flows (Δ*Q*) Guard-gap (*Q_gap_*) Auxiliary insulation (*Q*′) Edge effects (*Q*_ε_)

**Table 3:** Summary of Standard Uncertainty Components for Meter Area (*A*)

*X_i_*	*x_i_*	*c_i_*	*u* (*x_i_*)	Type	*c_i_* · *u* (*x_i_*)
*r_o_*	0.20282 m	0.63763 m	0.0000254 m	B	16.20 × 10^−6^ m^2^
*r_i_*	0.20371 m	0.64042 m	0.0000254 m	B	16.27 × 10^−6^ m^2^
α	23.6 × 10^−6^ K^−1^	3.8953 m^2^ · K	2.36 × 10^−6^ K^−1^	B	9.19 × 10^−6^ m^2^
Δ*T_mp_*	15 K	6.13 × 10^−6^ m^2^ · K^−1^	0.086 K	B	0.53 × 10^−6^ m^2^

**Table 4:** Summary of Replication Statistics for Uncertainty Component *u* (*L*_3_)

Nominal *L* (mm)	Day	Replicates	Within-day Average (m)	Within-day Standard Deviation (m)	*S_a_* (m)	*S_d_* (m)	*u* (*L*_3_) (m)
25.4	1	5	0.0254051	3.96 × 10^−6^			
	2	5	0.0254144	4.28 × 10^−6^			
	3	5	0.0254156	3.29 × 10^−6^			
	4	5	0.0254159	5.20 × 10^−6^			
					5.12 × 10^−6^	4.24 × 10^−6^	6.37 × 10^−6^
76.2	1	5	0.0762217	0.70 × 10^−6^			
	2	5	0.0762325	1.93 × 10^−6^			
	3	5	0.0762376	1.38 × 10^−6^			
	4	5	0.0762325	3.77 × 10^−6^			
					6.69 × 10^−6^	2.25 × 10^−6^	6.98 × 10^−6^
152.4	1	5	0.152405	3.68 × 10^−6^			
	2	5	0.152410	0.70 × 10^−6^			
	3	5	0.152411	3.45 × 10^−6^			
	4	5	0.152409	2.98 × 10^−6^			
					2.48 × 10^−6^	2.95 × 10^−6^	3.62 × 10^−6^
228.6	1	5	0.228578	10.79 × 10^−6^			
	2	5	0.228569	7.64 × 10^−6^			
	3	5	0.228582	2.75 × 10^−6^			
	4	5	0.228571	2.63 × 10^−6^			
					6.28 × 10^−6^	6.88 × 10^−6^	8.79 × 10^−6^

**Table 5:** Summary of Standard Uncertainty Components for 25.4 mm Thickness (*L*_25.4_)

*u* (*x_i_*)	Source	*c_i_*	Value of *u* (*L_i_*)	Type
*u* (*L*_1_)	In-situ measurement multiple observations system uncertainty	1	20 × 10^−6^ m	B
			19 × 10^−6^ m	A (degrees of freedom = 3)
			5.0 × 10^−6^ m	B (equipment specification, *k* = 1)

*u* (*L*_2_)	Spacers (nominal 25.4) repeated observations caliper uncertainty	1	1.9 × 10^−6^ m	B
			1.1 × 10^−6^ m	A (degrees of freedom = 12)
			1.5 × 10^−6^ m	B (a/√3 where a = 2.54 × 10^−6^ m)

*u* (*L*_3_)	Short-term repeatability	1	6.4 × 10^−6^ m	A (degrees of freedom = 6.8)

*u* (*L*_4_)	Plate flatness repeated observations CMM uncertainty	1	7.9 × 10^−6^ m	B
			2.3 × 10^−6^ m	A (degrees of freedom = 31)
			5.1 × 10^−6^ m	B (equipment specification, *k* = 1)

*u* (*L*_5_)	Plate deflection under load	1	31 × 10^−6^ m	B (calculation [15])

**Table 6:** Combined Standard Uncertainty *u_c_*(*L*)

(*L*) (mm)	*u* (*L*_1_) (m)	*u* (*L*_2_) (m)	*u* (*L*_3_) (m)	*u* (*L*_4_) (m)	*u* (*L*_5_) (m)	*u_c_* (*L*) (mm)	*u_c,r_*(*L*) (%)
25.4	20 × 10^−6^	1.9 × 10^−6^	6.4 × 10^−6^	7.9 × 10^−6^	31 × 10^−6^	0.038	0.15
76.2	12 × 10^−6^	2.4 × 10^−6^	7.0 × 10^−6^	7.9 × 10^−6^	31 × 10^−6^	0.035	0.05
152.4	12 × 10^−6^	7.7 × 10^−6^	3.6 × 10^−6^	7.9 × 10^−6^	31 × 10^−6^	0.035	0.02
228.6	9.6 × 10^−6^	9.5 × 10^−6^	8.8 × 10^−6^	7.9 × 10^−6^	31 × 10^−6^	0.035	0.02

**Table 7:** Summary of Standard Uncertainty Components for *T*

*u* (*x_i_*)	Source	*c_i_*	Value of *u* (*T_i_*)	Type
*u* (*T*_1_)	Measurement (*T_h_*, *T_c_*)	1	0.058 K	B
	DMM incertainty regression uncertainty	1	0.058 K	B ( $a/\surd \bar{3}$ where *a* = 0.039 Ω)
		1	0.0052 K	A (degrees of freedom = 15)

*u* (*T*_2_)	Calibration of PRTs	1	0.005 K	B (NIST Certificate, *k* = 1)

*u* (*T*_3_)	Miscellaneous	1	0.019 K	B
	Contact resistance	1	0.0017 K	B
	Sampling (planar)	1	0.015 K	B (Reference [4])
	Axial variation in plate	1	0.011 K	B (Reference [12])

*u_c_*(*T*)			0.061 K	

**Table 8:** Summary of Standard Uncertainty Components for Power Input (*Q_m_*) at *L* = 25.4 mm

*X_i_*	*x_i_*	*c_i_*	*u* (*x_i_*)	Type
*V_s_*	0.03 V	169 A	8.7 × 10^−6^ V	B ( $a/\surd \bar{3}$ where *a* = 15.0 μV)
*R_s_*	0.10006957 Ω	50.93 V^2^ · Ω^−2^	2.5 × 10^−7^ Ω	B (NIST Certificate, *k* = 1)
*V_m_*	17 V	0.3 A	1.76 × 10^−3^ V	B ( $a/\surd \bar{3}$ where *a* = 3.05 mV)

**Table 9:** Predicted values for *ε* due to Edge Heat Transfer (Peavy and Rennex [22])

*L* (mm)	A	*B*	*X* = −0.450	*ε* *X* = 0	*X* = +0.450
25.4	0	0	0	0	0
76.2	0	0.0000021	0	0	0
152.4	0.0000021	0.0022826	−0.0010	0	+0.0013
228.6	0.0002111	0.0266257	−0.0118	0.0002	+0.0122

**Table 10:** Nominal Settings for Imbalance Study in Yates Order

#	*V_gap_* (μV)	*T*′*_h_* − *T*′*_c_* (K)	*T_m_* − *T_a_* (K)
1	−50	−0.5	−5
2	+50	−0.5	−5
3	−50	+0.5	−5
4	+50	+0.5	−5
5	−50	−0.5	+5
6	+50	−0.5	+5
7	−50	+0.5	+5
8	+50	+0.5	+5
9	0	0	0

**Table 11a:** Imbalance Test Data in Yates Order

	25.4 mm					76.2 mm				

*#*	*Q_m_* (W)	Δ*T* (K)	*V_gap_* (μV)	*T*′*_h_* − *T*′*_c_* (K)	*T_m_* − *T_a_* (K)	*Q_m_* (W)	Δ*T* (K)	*V_gap_* (μV)	*T*′*_h_* − *T*′*_c_* (K)	*T_m_* − *T_a_* (K)
1	4.9701	22.22	−49.96	−0.501	−5.00	1.6137	22.22	−49.96	−0.499	−5.00
2	5.2257	22.22	+50.02	−0.505	−5.00	1.8798	22.22	+49.99	−0.495	−5.00
3	5.0159	22.22	−49.97	+0.499	−5.00	1.6621	22.22	−50.00	+0.499	−5.00
4	5.2768	22.22	+50.03	+0.502	−5.00	1.9285	22.22	+50.00	+0.497	−5.00
5	4.9589	22.22	−49.94	−0.503	+5.00	1.6165	22.22	−50.02	−0.500	+5.00
6	5.2153	22.22	+50.01	−0.498	+5.00	1.8829	22.22	+49.96	−0.500	+5.01
7	5.0067	22.22	−49.96	+0.506	+5.00	1.6644	22.22	−49.98	+0.497	+5.00
8	5.2652	22.22	+50.05	+0.501	+5.00	1.9306	22.22	+50.03	+0.497	+5.00
9	5.1189	22.23	0.06	0	0	1.7719	22.22	0.00	0	0

**Table 11b:** Imbalance Test Data in Yates Order

	152.4 mm					228.6 mm				

#	*Q_m_* (W)	Δ*T* (K)	*V_gap_* (μV)	*T*′*_h_* − *T*′*_c_* (K)	*T_m_* − *T_a_* (K)	*Q_m_* (W)	Δ*T* (K)	*V_gap_* (μV)	*T*′*_h_* − *T*′*_c_* (K)	*T_m_* − *T_a_* (K)
1	0.7276	22.22	−49.98	−0.500	−5.00	0.4425	22.22	−49.99	−0.502	−5.00
2	0.9978	22.22	+49.99	−0.502	−5.00	0.7225	22.22	+49.97	−0.499	−5.00
3	0.7752	22.22	−49.98	+0.495	−5.00	0.4915	22.22	−49.91	+0.499	−5.00
4	1.0461	22.22	+49.98	+0.493	−5.00	0.7714	22.21	+50.03	+0.503	−5.00
5	0.7269	22.23	−50.00	−0.500	+5.00	0.4397	22.22	−49.99	−0.503	+5.01
6	0.9975	22.22	+50.04	−0.506	+5.00	0.7200	22.22	+50.07	−0.492	+5.00
7	0.7757	22.21	−50.06	+0.497	+5.01	0.4897	22.22	−49.97	+0.500	+5.00
8	1.0457	22.22	+50.02	+0.500	+5.00	0.7660	22.22	+49.96	+0.495	+5.01
9	0.8852	22.22	+0.01	0	0	0.6042	22.21	+0.03	0	0

**Table 12:** Statistical Significance for Estimated Effects for Imbalance Study

Factor	25.4 mm	76.2 mm	152.4 mm	228.6 mm
*x*	**	**	**	**
*x*_2_	*	**	**	*
*x*_3_	*	*	—	—
*x*_1_*x*_2_	—	—	—	—
*x*_1_*x*_3_	—	—	—	—
*x*_2_*x*_3_	—	—	—	—
*x*_1_*x*_2_*x*_3_	—	—	—	—

** Statistically significant at the l % level

* Statistically significant at the 5 % level

**Table 13:** Estimates and Standard Deviations for *a*_1_, *a*_2_, and *a*_3_ in Eq. (24)

*L* (mm)	*a*_l_ (W·μV^−1^)	*s*(*a*_l_) (W·μV^−1^)	*a*_2_ (W·K^−1^)	*s*(*a*_2_) (W·K^−1^)	*a*_3_ (W·K^−1^)	*s*(*a*_3_) (W·K^−1^)	RSD* (W)
25.4	+0.002579	2.15 × 10^−5^	0.04846	2.14 × 10^−3^	+0.001072	2.15 × 10^−4^	0.0030
76.2	+0.002663	4.59 × 10^−6^	0.04833	4.61 × 10^−4^	−0.000269	4.59 × 10^−5^	0.0006
152.4	+0.002705	1.36 × 10^−5^	0.04836	1.36 × 10^−3^	+0.000028	1.36 × 10^−4^	0.0019
228.6	+0.002790	1.24 × 10^−5^	0.04856	1.24 × 10^−3^	+0.000309	1.24 × 10^−4^	0.0017

* Residual standard deviation for fit

**Table 14:** Comparison of Empirical and Theoretical Values of Edge Heat Flow Error (*ε*)

*L* (mm)	Empirical *ε* (Equation 27))			Theoretical *ε* (Table 9)		
	*T_m_* − *T_a_* = −5 K	*T_m_* − *T_a_* = 0 K	*T_m_* − *T_a_* = +5 K	*X* = − 0.450	*X* = 0	*X* = + 0.450
25.4	−0.0010	0	+0.0010	0	0	0
76.2	+0.0008	0	−0.0008	0	0	0
152.4	−0.0001	0	+0.0001	−0.0010	0	+0.0013
228.6	−0.0025	0	+0.0025	−0.0118	0.0002	+0.0122

**Table 15:** Estimates for *u_c_* (Δ*Q*)

*L* (mm)	*x*_l_ (μV)	*u*(*a*_l_) (W·μV^−1^)	*a*_l_ (W·μV^−1^)	*u*(*x*_l_) (K)	*x*_2_ (K)	*u*(*a*_2_) (W·K^−1^)	*a*_2_ (W·K^−1^)	*u*(*x*_2_) (K)	*x*_3_ (K)	*u*(*a*_3_) (W·K^−1^)	*a*_3_ (W·K^−1^)	*u*(*x*_3_) (K)	*u_c_*(Δ*Q*) (W)
25.4	0.01	2.15 ×10^−5^	+0.002579	2.48	0.005	2.14 ×10^−3^	0.04846	0.086	0.004	2.15 ×10^−4^	+0.001072	0.5	0.0087
76.2	0.02	4.59 × 10^−6^	+0.002663	2.48	0.003	4.61 × 10^−4^	0.04833	0.086	0.003	4.59 × 10^−5^	−0.000269	0.5	0.0082
152.4	0.01	1.36 ×10^−5^	+0.002705	2.48	0.002	1.36 ×10^−3^	0.04836	0.086	0.004	1.36 ×10^−4^	+0.000028	0.5	0.0088
228.6	0.01	1.24 × 10^−5^	+0.002790	2.48	0.002	1.24 × 10^−3^	0.04856	0.086	0.002	1.24 × 10^−4^	+0.000309	0.5	0.0083

**Table 16:** Combined Standard Uncertainty *u_c (_Q*)

*L* (mm)	*Q* (W)	*u_A_*(*Q_m_*) (W)	*u_B_*(*Q_m_*) (W)	*u_c_*(Δ*Q*) (W)	*u_c_*(*Q*) (W)	*u_c,r_*(*Q*) (%)
25.4	5.1452	0.0006	0.0016	0.0087	0.0089	0.17
76.2	1.8032	0.0004	0.0007	0.0082	0.0082	0.46
152.4	0.8761	0.0003	0.0004	0.0088	0.0088	1.0
228.6	0.6112	0.0003	0.0003	0.0083	0.0084	1.4

**Table 17a:** Combined Standard Uncertainty for Thermal Conductivity (*λ*_exp_) for *L* = 25.4 mm

*X_i_*	*x_i_*	*c_i_*	*u*(*x_i_*)	|*c_i_ u*| (W·m^−1^·K^−1^)	(*c_i_ u*)/*y* (%)
*Q*	5.1452 W	0.00881 m^−1^·K^−1^	0.0089 W	0.00008	0.17
*L*	0.0254 m	1.78291 W·m^−2^·K^−1^	3.8 × 10^−5^ m	0.00007	0.15
*A*	0.12989 m^2^	−0.34883 W·m^−3^·K^−1^	2.47 × 10^−5^ m^2^	0.00001	0.02
Δ*T*	22.22 K	−0.00204 W·m^−1^·K^−2^	0.086 K	0.00017	0.39

*y* = *λ*_exp_ = 0.0454 W·m^−1^·K^−1^(*ρ* = 9.4 kg·m^−3^)			*u_c_*(*λ*_exp_)	0.00020	0.45
	
			*U*(*k* = 2)	0.00041	0.9

**Table 17b:** Combined Standard Uncertainty for Thermal Conductivity (*λ*_exp_) for *L* = 76.2 mm

*X_i_*	*x_i_*	*c_i_*	*u*(*x_i_*)	|*c_i_ u*| (W·m^−1^·K^−1^)	(*c_i_ u*)/*y* (%)
*Q*	1.8032 W	0.02641 m^−1^·K^−1^	0.0082 W	0.00022	0.46
*L*	0.0762 m	0.62466 W·m^−2^·K^−1^	3.5 × 10^−5^ m	0.00002	0.05
*A*	0.12989 m^2^	−0.36657 W·m^−3^·K^−1^	2.47 × 10^−5^ m^2^	0.00001	0.02
Δ*T*	22.22 K	−0.00214 W·m^−1^·K^−2^	0.086 K	0.00018	0.39

*y* = *λ*_exp_ = 0.0477 W·m^−1^·K^−1^(*ρ* = 9.0 kg·m^−3^)			*u_c_*(*λ*_exp_)	0.00029	0.60
	
			*U*(*k* = 2)	0.00057	1.2

**Table 17c:** Combined Standard Uncertainty for Thermal Conductivity (*λ*_exp_) for *L* = 152.4 mm

*X_i_*	*x_i_*	*c_i_*	*u*(*x_i_*)	|*c_i_ u*| (W·m^−1^·K^−1^)	(*c_i_ u*)/*y* (%)
*Q*	0.8761 W	0.05280 m^−1^·K^−1^	0.0088 W	0.00046	1.0
*L*	0.1524 m	0.30356 W·m^−2^·K^−1^	3.5 × 10^−5^ m	0.00001	0.02
*A*	0.12989 m^2^	−0.35614 W·m^−3^·K^−1^	2.47 × 10^−5^ m^2^	0.00001	0.02
Δ*T*	22.22 K	−0.00208 W·m^−1^·K^−2^	0.086 K	0.00018	0.39

*y* = *λ*_exp_ =0.0463 W·m^−1^·K^−1^(*ρ* = 9.6 kg·m^−3^)			*u_c_*(*λ*_exp_)	0.00049	1.1
	
			*U*(*k* = 2)	0.00099	2.1

**Table 17d:** Combined Standard Uncertainty for Thermal Conductivity (*λ*_exp_) for *L* = 228.6 mm

*X_i_*	*x_i_*	*c_i_*	*u*(*x_i_*)	|*c_i_ u*| (W·m^−1^·K^−1^)	(*c_i_ u*)/*y* (%)
*Q*	0.6112 W	0.07920 m^−1^·K^−1^	0.0084 W	0.00066	1.4
*L*	0.12286 m	0.21178 W·m^−2^·K^−1^	3.5 × 10^−5^ m	0.00001	0.02
*A*	0.12989 m^2^	−0.37270 W·m^−3^·K^−1^	2.47 × 10^−5^ m^2^	0.00001	0.02
Δ*T*	22.22 K	−0.00218 W·m^−1^·K^−2^	0.086 K	0.00019	0.39

*y* = *λ*_exp_ = 0.0484 W·m^−1^·K^−1^(*ρ* = 8.7 kg·m^−3^)			*u_c_*(*λ*_exp_)	0.00068	1.4
	
			*U*(*k* = 2)	0.00137	2.8

**Table 18a:** Combined Standard Uncertainty for Thermal Resistance (*R*) for *L* = 25.4 mm

*X_i_*	*x_i_*	*c_i_*	*u*(*x_i_*)	|*c_i_ u*| (m^2^·K·W^−1^)	(*c_i_ u*)/*y* (%)
*Q*	5.1452 W	−0.10901 m^2^·K·W ^−2^	0.0089 W	0.00097	0.17
*A*	0.12989 m^2^	4.31811 K·W^−1^	2.47 × 10^−5^ m^2^	0.00011	0.02
Δ*T*	22.22 K	0.02524 m^2^·W^−1^	0.086 K	0.00218	0.39

*y* = *R* = 0.560 m^2^·K·W^−1^			*u_c_*(*R*)	0.0024	0.43
	
			*U*(*k* = 2)	0.0048	0.9

**Table 18b:** Combined Standard Uncertainty for Thermal Resistance (*R*) for *L* = 76.2 mm

*X_i_*	*x_i_*	*c_i_*	*u*(*x_i_*)	|*c_i_ u*| (m^2^·K·W^−1^)	(*c_i_ u*)/*y* (%)
*Q*	1.8032 W	−0.88782 m^2^·K·W^−2^	0.0082 W	0.00732	0.46
*A*	0.12989 m^2^	12.325 K·W^−1^	2.47 × 10^−5^ m^2^	0.00030	0.02
Δ*T*	22.22 K	0.07204 m^2^·W^−1^	0.086 K	0.00621	0.39

*y* = *R* = 1.60 m^2^·K·W^−1^			*u_c_*(*R*)	0.010	0.60
	
			*U*(*k* = 2)	0.020	1.2

**Table 18c:** Combined Standard Uncertainty for Thermal Resistance (*R*) for *L* = 152.4 mm

*X_i_*	*x_i_*	*c_i_*	*u*(*x_i_*)	|*c_i_ u*| (m^2^·K·W^−1^)	(*c_i_ u*)/*y* (%)
*Q*	0.8761 W	−3.7599 m^2^·K·W^−2^	0.0088 W	0.03295	1.0
*A*	0.12989 m^2^	25.3613 K·W^−1^	2.47 × 10^−5^ m^2^	0.00063	0.02
Δ*T*	22.22 K	0.14825 m^2^·W^−1^	0.086 K	0.01278	0.39

*y* = *R* = 3.29 m^2^·K·W^−1^			*u_c_*(*R*)	0.036	1.1
	
			*U*(*k* = 2)	0.071	2.2

**Table 18d:** Combined Standard Uncertainty for Thermal Resistance (*R*) for *L* = 228.6 mm

*X_i_*	*x_i_*	*c_i_*	*u*(*x_i_*)	|*c_i_ u*| (m^2^·K·W^−1^)	(*c_i_ u*)/*y* (%)
*Q*	0.6112 W	−7.72508 m^2^·K·W^−2^	0.0084 W	0.06454	1.4
*A*	0.12989 m^2^	36.3531 K·W^−1^	2.47 × 10^−5^ m^2^	0.00090	0.02
Δ*T*	22.22 K	0.21250 m^2^·W^−1^	0.086 K	0.01832	0.39

*y* = *R* = 4.72 m^2^·K·W^−1^			*u_c_*(*R*)	0.0068	1.4
	
			*U*(*k* = 2)	0.135	2.8

**Table 19:** Typical Values of *R* and *U_r_* for Low-Density Fibrous-Glass Blanket N1ST CTS

*L* (mm)	*R* (m^2^·K·W^−1^)	*U_r_* (%)
25.4	0.564	1.0
76.2	1.61	1.5
152.4	3.31	2.5
28.6	4.75	3.0

**Table 20:** Percent Contribution of Individual Components for *λ*_exp_ and *R*

*λ*_exp_ (Table 17)					*R* (Table 18)			
*X_i_*	25.4 mm	76.2 mm	152.4 mm	228.6 mm	25.4 mm	76.2 mm	152.4 mm	228.6 mm
*A*	0.02	0.02	0.02	0.02	0.02	0.02	0.02	0.02
*L*	0.15	0.05	0.02	0.02	—	—	—	—
Δ*T*	0.39	0.39	0.39	0.39	0.39	0.39	0.39	0.39
*Q*	0.17	0.46	1.0	1.4	0.17	0.46	1.0	1.4

**Table 21:** Comparison of Individual Components in (%) for *λ*_exp_

*λ*_exp_ (Table 17)					*λ*_exp_ (from Table 1 in Ref. [4])			
*X_i_*	25.4 mm	76.2 mm	152.4 mm	228.6 mm	25 mm	75 mm	150 mm	300 mm
*A*	0.02	0.02	0.02	0.02	0.01	0.01	0.01	0.01
*L*	0.15	0.05	0.02	0.02	0.1	0.03-	0.02	0.01
Δ*T*	0.39	0.39	0.39	0.39	0.16	0.16	0.16	0.16
*Q*	0.17	0.46	1.0	1.4	0.04*	0.06*	0.08*	0.61*

* Value obtained by summing heat flow estimates from Table 1 in Ref. [4].

## Footnotes

1 Certain commercial entities, equipment, or materials may be identified in this document in order to describe an experimental procedure or concept adequately. Such identification is not intended to imply recommendation or endorsement by the National Institute of Standards and Technology, nor is it intended to imply that the entities, materials, or equipment are necessarily the best available for the purpose.

2 In 1901, Congress established the National Bureau of Standards (NBS) to support industry, commerce, scientific institutions, and all branches of government. In 1988, as part of the Omnibus Trade and Competiveness Act, the name was changed to the National Institute of Standards and Technology (NIST) to reflect the agency′s broader mission. For historical accuracy, this report uses, where appropriate, NBS for events prior to 1988.

3 The thermal transmission properties of heat insulators determined from standard test methods typically include several mechanisms of heat transfer, including conduction, radiation, and possibly convection. For that reason, some experimentalists will include the adjective “apparent” when describing thermal conductivity of thermal insulation. However, for brevity, the term thermal conductivity is used in this report.

4 Terminology for the 1016 mm guarded hot plate reflects current usage in ASTM Practice C 1043.

5 Type E is a letter designation for an ANSI standard base-metal thermocouple. Thermoelectric elements are designated by two letters where the second letter, P or N, denotes the positive or negative thermoelement, respectively.

6 Note that the probability distribution for a Type B evaluation, in contrast to a Type A evaluation, is assumed based on the judgment of the experimenter.

7 For a two-sided test (Fig. 1), eight linear positioning devices (four for each specimen) determine the in-situ thickness of the specimen pair.

8 Rennex [4] obtained a value of approximately 0.00057 W·μV^−1^ for an 18-pair Type E thermopile used in the guard gap. In 1994, the 18-pair thermopile was replaced, as part of upgrade and regular maintenance, with the 4-pair Type E thermopile presented in this report. Note that the difference in sensitivities between the 4-pair and 18-pair thermopiles is 4.5, roughly the same ratio observed between the value reported by Rennex [4] and the values for *a*_1_ reported in Table 13 (0.0026/0.00057 = 4.56).

## Appendix A Meter Area (*A*) Derivation

Test Method C 177 [7] defines the meter area as:

(A-1) $$A=A_{mp}+\frac{A_{\mathrm{gap}}}{2}$$

where:

*A_mp_* = surface area of the meter plate (m^2^); and,
*A_gap_* = surface area of guard gap between meter plate and the guard plate (Figs. 1 and 2).

For the circular meter area of the NIST guarded-hotplate, Eq. (A-1) is rewritten as

(A-2) $$A=\pi r_{o}^{2}+(\pi r_{i}^{2}-\pi r_{o}^{2})/2=\frac{\pi }{2}(r_{o}^{2}+r_{i}^{2})$$

where:

*r_o_* = outer radius of meter plate (m); and,
*r_i_* = inner radius of guard plate (m).

Including thermal expansion effects, Eq. (A-2) is rewritten in the following form:

(A-3) $$\begin{array}{l} A=\frac{\pi }{2}{\left[r_{0}^{2}(1+a\Delta T_{mp})+r_{i}^{2}(1+a\Delta T_{mp})\right]}^{2}\\ \;=\frac{\pi }{2}\left[(r_{o}^{2}+r_{i}^{2}){\left(1+a\Delta T_{mp}\right)}^{2}\right] \end{array}$$

where:

*a* = coefficient of thermal expansion of aluminum (alloy 6061-T6) (K^−l^); and,
Δ*T_mp_* = temperature difference of the meter plate from ambient (K) = *T_h_* − 20 °C.

Further simplification of Eq. (A-3) can be obtained as shown in Eq. (A-4):

(A-4) $$A=\pi r_{c}^{2}{\left(1+a\Delta T\right)}^{2}$$

by substitution of:

$$r_{c}=\sqrt{\frac{r_{o}^{2}+r_{i}^{2}}{2}.}$$
